# Single-nucleotide-resolution mapping of DNA gyrase cleavage sites across the *Escherichia coli* genome

**DOI:** 10.1093/nar/gky1222

**Published:** 2018-12-04

**Authors:** Dmitry Sutormin, Natalia Rubanova, Maria Logacheva, Dmitry Ghilarov, Konstantin Severinov

**Affiliations:** 1 Centre for Life Sciences, Skolkovo Institute of Science and Technology, 143026 Moscow, Russia; 2 Department of Bioengineering and Bioinformatics, Lomonosov Moscow State University, 119991 Moscow, Russia; 3 Malopolska Centre of Biotechnology, Jagiellonian University, 30387 Cracow, Poland; 4 Waksman Institute for Microbiology, Rutgers, The State University of New Jersey, Piscataway, NJ 08854, USA

## Abstract

An important antibiotic target, DNA gyrase is an essential bacterial enzyme that introduces negative supercoils into DNA and relaxes positive supercoils accumulating in front of moving DNA and RNA polymerases. By altering the superhelical density, gyrase may regulate expression of bacterial genes. The information about how gyrase is distributed along genomic DNA and whether its distribution is affected by drugs is scarce. During catalysis, gyrase cleaves both DNA strands forming a covalently bound intermediate. By exploiting the ability of several topoisomerase poisons to stabilize this intermediate we developed a ChIP-Seq-based approach to locate, with single nucleotide resolution, DNA gyrase cleavage sites (GCSs) throughout the *Escherichia coli* genome. We identified an extended gyrase binding motif with phased 10-bp G/C content variation, indicating that bending ability of DNA contributes to gyrase binding. We also found that GCSs are enriched in extended regions located downstream of highly transcribed operons. Transcription inhibition leads to redistribution of gyrase suggesting that the enrichment is functionally significant. Our method can be applied for precise mapping of prokaryotic and eukaryotic type II topoisomerases cleavage sites in a variety of organisms and paves the way for future studies of various topoisomerase inhibitors.

## INTRODUCTION

DNA supercoiling accompanies processes that involve unwinding of double helix, i.e. transcription, replication and recombination (1–4). Topoisomerases (topos) control the level of DNA supercoiling, resolve entangled DNA structures (knots and catenanes), and may take part in genome compaction (4–6). Depending on the mechanism of their action, two major types of topo enzymes are distinguished (7). A type II topo present in bacteria, DNA gyrase, is the only known enzyme that can introduce negative supercoils using the chemical energy of ATP hydrolysis (8). Gyrase, together with another type II topo operating in *Escherichia coli*, Topo IV, is indispensable for DNA replication. Current view is that Topo IV acts on pre-catenanes forming behind the fork while gyrase is well suited to relax positive supercoils accumulating in front of the replisome (9,10). In *E. coli*, a balance between type I Topo I relaxation activity and DNA gyrase supercoiling activity is required to achieve and maintain superhelical density levels optimal for different physiological states (11–16).

The distribution of topoisomerases across the chromosome is thought to be governed, in part, by transcription-induced supercoiling. The ‘twin supercoiled-domain model’ proposed by Liu and Wang in 1987 (17) explained the earlier observation that transcription affected supercoiling (18) and was confirmed experimentally a year later (19). It envisions that transcribing RNA polymerase (RNAP) generates downstream positive supercoils, while the same number of negative supercoils is formed upstream. DNA gyrase has an increased affinity for positively supercoiled regions (20) and, according to the model, relaxes DNA downstream of the transcription elongation complex, while Topo I, which prefers negatively supercoiled DNA (21,22), acts upstream. Recent genome-wide studies using ChIP-Seq in *Mycobacterium tuberculosis* (23) and ChIP-chip in *E. coli* (24) reveal that global distribution of gyrase and Topo I is generally consistent with the ‘twin supercoiled-domain model’ expectations. Specifically, the Ori-Ter gradient of gyrase binding (24–26) is thought to correspond to the gradient of transcription activity, which leads to positive supercoiling neutralization in the *E. coli* genome during the exponential phase of growth (27). However, it is still unknown whether gyrase-binding gradient is the intrinsic property of the genome in terms of the binding sites location, or is caused by transcription-mediated supercoiling.

Gyrase preferentially binds some sequences (strong gyrase sites, SGSs) irrespective of transcription. The best-studied Mu SGS is essential for replication of *E. coli* bacteriophage Mu; it allows organization of the integrated viral genome into a separate topological domain leading to efficient recombination of aligned prophage ends (28–30). Genetic analysis of Mu SGS highlighted the importance of anisotropically flexible right arm for gyrase binding (31,32). Other SGSs were found in the pSC101 and pBR322 plasmids (33,34). Gyrase was also shown to preferentially bind and cleave bacterial interspersed mosaic elements (BIMEs) in the *E. coli* genome (35,36).

**Figure 1.**
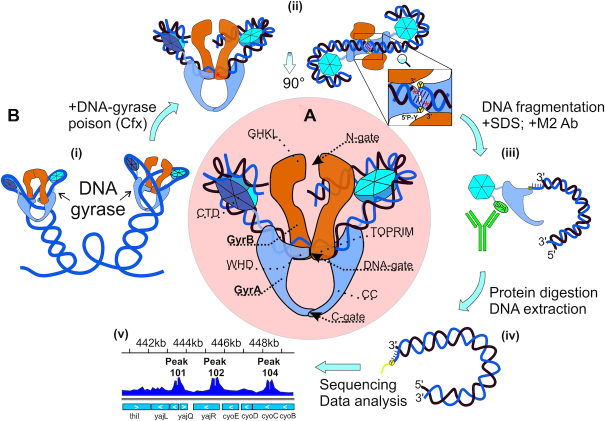
DNA gyrase structure and Topo-Seq procedure. (**A**) DNA gyrase structure with gyrase domains (GHKL, TOPRIM, WHD, CC, CTD) indicated. (**B**) Topo-Seq workflow: (i) Schematic illustration of DNA gyrase on DNA plectonemes; (ii) A gyrase-DNA complex trapped with an inhibitor, here quinolone-like (red stars); (iii) C-terminal SPA tag is recognized by M2 antibodies and used to precipitate gyrase-bound fragments; (iv) Deproteinized DNA fragments have blocked 5′-ends; (v) Resulting signals visualized.

DNA gyrase functions as a GyrA_2_GyrB_2_ tetramer with three major interfaces called ‘gates’. The gates can temporarily open, allowing transfer of double-stranded DNA (Figure 1A). The ‘N-gate’ is formed by the N-terminal ATP-binding GHKL domains of GyrB; the ‘DNA-gate’ is formed by the C-terminal TOPRIM domain of GyrB and the N-terminal WHD domain of GyrA, while the ‘C-terminal’ or ‘exit’ gate is formed by coiled-coiled (CC) domains of GyrA. Being a type II enzyme, gyrase introduces a double-strand break in the ‘G’ or ‘gate’ segment DNA bound at the GyrA dimer interface, forming a covalent intermediate cleavage complex. Upon the binding of ATP, the enzyme captures a ‘T’ or ‘transported’ DNA segment and transfers it through the break in the G segment. The double-strand break is next religated. The T segment must be located *in cis* with the G segment and the bound DNA must be wrapped around the enzyme forming a positive node. Wrapping is facilitated by the C-terminal domains of GyrA (CTDs) (Figure 1A). When the enzyme completes its cycle, two negative supercoils are introduced into a DNA molecule (37,38).

In the intermediate cleavage complex catalytic tyrosines of the GyrA subunits (Tyr122 in *E. coli* GyrA) with the help of Mg^2+^ ions coordinated by the TOPRIM domains of GyrB attack and cleave the G segment, forming 4-bp 5′-overhangs covalently linked via phosphodiester bonds to the tyrosine residues (39,40). Many gyrase inhibitors trap covalent cleavage complexes leading to accumulation of double-stranded breaks and, ultimately, cell death (41–44). Purification of trapped complexes followed by deproteination and sequencing (or hybridization) of recovered DNA allows one to analyze the sites of gyrase cleavage (Figure 1B). Earlier such approach was widely used for mapping of particular cleavage sites (29,33,45,46) and for investigating relatively short DNA regions in detail (34–36,47,48). Next-generation sequencing allowed genome-wide analysis: cleavage maps were generated for Topo IV in *E. coli* and Topo IIA in human cells (49,50). As a consequence of trapping, several amino acid residues from topoisomerase active site remain linked to the 5′-ends of nucleic acid fragments after proteolytic treatment, resulting in poor adapter ligation efficiency (49). To overcome this challenge, we developed a procedure, which we name Topo-Seq, that employs single-strand DNA sequencing protocol (51) to obtain information on DNA strands that remain free within the cleavage complexes. Since cleavage by gyrase generates 4-bp 5′-overhangs, application of our procedure results in 4-bp gaps in coverage at the cleavage sites, allowing their precise identification. We used Topo-Seq to generate whole-genome maps of gyrase cleavage events in *E. coli* induced by three different gyrase poisons: closely related ciprofloxacin/oxolinic acid and microcin B17. The latter has a different mechanism of action, giving us an opportunity to cross-validate the results and exclude possible drug-specific artifacts. We show that global gyrase distribution along the genome is governed by two factors: transcription intensity and direction, and binding preference for an extensive degenerate motif.

## MATERIALS AND METHODS

### Bacterial strains

*Escherichia coli* DY330 GyrA-SPA (W3110 Δ*lacU169 gal490* λ*cI857* Δ(cro-bioA) *gyrA*-SPA) was purchased from Dharmacon. *Escherichia coli* DY330 GyrA-SPA Mu SGS (DY330 *gyrA*-SPA (*dcuC-crcA*)::(*cat-Mu SGS*)) constructed for this study (strain construction details are described in one of the following sections of Materials and Methods) were used for Topo-Seq and Topo-qPCR experiments and MIC estimation. Both strains have *gyrA* gene fused with C-terminal SPA affinity tag for efficient purification of GyrA subunits. For Topo-Seq experiments bacteria were inoculated from glycerol stocks stored at −80°C on agar plates containing corresponding antibiotics (kanamycin 50 μg/ml for DY330 GyrA-SPA and kanamycin 50 μg/ml, chloramphenicol 15 μg/ml for DY330 GyrA-SPA Mu SGS). Plates were incubated at 32°C for 24–32 h, then stored at 4°C for 1 week. Liquid cultures were started from single isolated colonies and cultivated in 2YT medium at 32°C with shaking (180rpm). *E. coli* BW25113 (52) was used for microcin B17 production as described in (53). *Escherichia coli* NEB5α was used for plasmids production for *in vitro* experiments.

### Microcin B17 purification

*Escherichia coli* BW25113 was transformed with *pBAD-mcbABCDEFG* plasmid and night culture was inoculated with one isolated colony. One liter of 2YT media was inoculated with 1/100 volume of the overnight culture. When the culture reached OD_600_ = 0.6–0.8, *mcb* operon expression was induced by addition of arabinose up to 1 mM. Cultivation was continued for 18–20 h on 37°C with shaking on 180 rpm. Cells were pelleted by centrifugation, then resuspended in 40 ml of 100 mM acetic acid/1 mM EDTA and boiled for 15 min. The clarified supernatant was applied onto 1 g C18 HyperSep cartridge (Thermo Scientific) pre-equilibrated with 0.1% trifluoroacetic acid (TFA). The cartridge was extensively washed with 0.1% TFA followed by 10% acetonitrile (ACN) in 0.1% TFA. The microcin B17-containing fraction was eluted in 30 ml 30% ACN in 0.1% TFA and vacuum dried (GeneVac). The resulting precipitate was dissolved in dimethyl sulfoxide (DMSO) and applied onto Phenomenex Luna C18 high-performance liquid chromatography (HPLC) column (pre-equilibrated with 0.1% TFA) in 10% DMSO/0.1% TFA. Elution was performed with linear gradient of ACN (from 0% to 50% ACN in 30 min) in 0.1% TFA. Microcin B17 was eluted between 12 and 16 min, individual peaks were collected. Fractions obtained were merged and dried *in vacuo*. Lyophilized powder was dissolved in DMSO and stored at −20°C. Concentration of the microcin B17 was determined spectroscopically as described previously (54).

### *E. coli D*Y 330 *gyrA-SPA Mu SGS* strain construction

349-bp DNA fragment containing strong gyrase binding site from bacteriophage Mu (Mu SGS) was amplified from pMP1000 plasmid (45) (a gift of P. Higgins) using Mu_G_F and Mu_G_R primers. *cat* gene was amplified from pKD3 plasmid (52) with G_cat_Mu and cat_Mu_R primers (Supplementary Table S2). Fragments were joined by overlap PCR using G_cat_Mu and Mu_G_R primers. Resulting cassette was inserted into intergenic region between *dcuC* and *crcA* genes using recombination techniques described elsewhere (55). Successful insertion was confirmed by PCR and whole genome sequencing.

### Minimal inhibitory concentration (MIC) measurement

MICs were determined by microdilution method in 96-well plates in liquid LB according to CLSI guidelines (M07-A10). Inoculum suspensions were prepared by dilution of night cultures grown in LB up to 1–5 × 10^5^ CFU/ml. Plates were incubated 18–24 h on 37°C, MIC recorded as minimal concentration of antibiotic that completely inhibit bacterial growth.

### ChIP with DNA gyrase poisons as stabilizing agents (Topo-Seq step I)

One milliliter of overnight culture was prepared for *E. coli* DY330 GyrA-SPA or *E. coli* DY330 GyrA-SPA Mu SGS by inoculating 2YT medium supplemented with antibiotics (kanamycin 50 μg/ml for DY330 GyrA-SPA and kanamycin 50 μg/ml, chloramphenicol 15 μg/ml for DY330 GyrA-SPA Mu SGS) with cells from a single colony. The culture was cultivated at 32°C with shaking (180 rpm) and then inoculated into 100 ml of 2YT without antibiotics and cultivation was continued at 37°C and shaking until culture reaching mid-logphase (OD_600_ = 0.6–0.8). At this point, the culture was bisected and DNA gyrase poison (0.9 or 10 μM ciprofloxacin, 120 μM oxolinic acid, 10 or 50 μM microcin B17—see Supplementary Table S1 for details) was added to the first half (+A samples), while the second served as a control (–A samples). Cultures (+A and –A) were incubated at 37°C with shaking for additional 15 min, then cells were pelleted by centrifugation at 10°C (4500 g) and resuspended in 10 ml of TES buffer (10 mM Tris–Cl pH 7.5, 1 mM EDTA, 250 mM NaCl). Washing procedure was repeated twice to remove all traces of culturing medium. Washed pellets were resuspended in 1 ml of TESS buffer (10 mM Tris–Cl pH 7.5, 1 mM EDTA, 250 mM NaCl, 0.02% SDS, 0.2% Tween-20) with addition of protease inhibitors cocktail (cOmplete ultra EDTA free, Roche) and RNAse A (Thermo Scientific). Resulting suspensions were sonicated with parameters optimized to obtain DNA fragments between 200 and 700 bp (24 cycles of 10 sec ON/20 sec OFF, 65% power, SONOPULS HD 3100). Lysates were diluted with 1 ml of TES buffer and 100 μl of ANTI-FLAG^®^ M2 affinity gel (Sigma-Aldrich) was added. Immunoprecipitation was performed for 1.5–2 h at room temperature with moderate mixing, then affinity gel was washed 4 times (two times with 1 ml of TESS buffer, once with 1 ml of TES buffer, and once 1 ml of TE buffer). For proteolysis, affinity gel obtained after the last wash step was diluted with TES buffer up to 200 μl, proteinase K (Sigma-Aldrich) was added (0.5 mg/ml) and samples were incubated at 55°C for at least 3 h. After this step samples were centrifuged (2 min, 2000g at room temperature) and DNA was extracted from resulting supernatant with phenol/chlorophorm method followed by ethanol precipitation. Mock controls (–IP) were made both for +A and for –A: for this, 100 μl aliquots of lysates obtained after sonication were deproteinized and DNA was purified as described before. The procedure described gives a quartet of samples (+A+IP, +A-IP, -A+IP, –A-IP), where +A-IP, –A+IP and –A-IP serves as controls for gyrase poison action and immunoprecipitation.

### DNA sequencing (Topo-Seq step II), reads trimming and alignment

Sequencing libraries were prepared with Accel NGS 1S kit (Swift Bioscience) from DNA obtained in step I procedure, according to the manufacturer's protocol. Sequencing was performed on Illumina NextSeq platform (150 bp paired-end reads) at A.N. Belozersky Research Institute of Physico-Chemical Biology MSU. Combination of gyrase poison-mediated ChIP procedure (Step I) and specific sequencing libraries preparation step (Step II) was named by us a Topo-Seq technique. For each antibiotic Topo-Seq was performed in triplicate.

Reads were aligned to the *E. coli* W3110 Mu SGS genome (*E. coli* W3110 genome with the insertion of *cat*-Mu SGS cassette may be downloaded from GEO: GSE95567) using BWA-MEM (56). BAM files were prepared with Samtools (57) and visualized in IGV (58).

### Coverage normalization and GCSs-calling procedure

For each position in the genome a number of 3′-ends (N3E) and 5′-ends were counted based on reads alignments stored in SAM file. Obtained values were divided by the total amount of reads aligned and multiplied by the lowest value across samples forming the quartet. Additional normalization was performed to get rid of the bias in the coverage depth across the genome: due to active replication, there is a significant difference in the total amount of DNA between origin of replication and terminators area. For this purpose, N3E values of +A+IP sample were divided by corresponding N3Es of +A-IP control and N3Es of –A+IP sample were divided by N3Es of –A-IP control (all samples originate from the same quartet). N3Es of +A-IP and –A-IP controls were preliminarily smoothed using 200 kb sliding window. In resulting pairs of samples (+A+IP_norm and –A+IP_norm) gyrase cleavage sites (GCSs) were called if values in *i* and *i* + 5 positions in +A+IP_norm sample both exceed the right confident interval value calculated based on the appropriate values in –A+IP sample (Audic and Claverie statistical test from (59), *P*-value < 0.05). As Topo-Seq was performed for each antibiotic in triplicate, GCS was called reliable if it was identified in at least two biological replicas. Only reliable GCSs sets were used for further analysis.

### qPCR validation of Topo-Seq (Topo-qPCR)

qPCR was performed to estimate the enrichment of DNA at specific loci after Step I (Mu SGS, rRNA A DS, ccmH and rRNA A US) and validate data obtained with Topo-Seq (primers listed in Supplementary Table S2). Step I of the procedure followed by qPCR was named Topo-qPCR.

### Gyrase motif identification

DNA sequences were extracted from *E. coli* W3110 Mu SGS genome as a 130 bp vicinity of identified GCSs’ positions. Therefore, all the sequences are centered relative to the DNA-gyrase cleavage sites and sequences sets can be processed as multiple alignments: nucleotide frequencies were counted within formed columns giving position probability matrix (PPM) visualized with Python Matplotlib package (60). The degenerate GC% motif was obtained similarly by calculating the frequency of G or C in the columns of the alignment. Logos were calculated with WebLogo for the same sets of DNA sequences (61). Motif visualization and Logos were made for each Topo-Seq condition independently. ‘Combined’ gyrase motif was constructed using sequences obtained from Cfx, Micro and Oxo Topo-Seq experiments. For each antibiotic top 732 GCSs having the highest N3E values were taken, resulting in a set containing 1828 sequences that was used for PPM and Position Weight Matrix construction. Antibiotic-specific bias was removed for positions most influenced by antibiotics (0–3 bp; in coordinates we use, cleavage takes place between positions -1/0 and +3/+4) in the intermediate PPM by changing corresponding values with a baseline frequencies of nucleotides observed in a *E. coli* W3110 genome. PPM can be found in Supplementary Table DS10. To find potential DNA gyrase binding sites, sequences of interest were scanned with final PWM in forward and reverse-complement forms. For the particular position, a maximum between values obtained for both strands was specified and referred further as “score”. PWM construction and sequences scanning were performed with Bio.motifs from Biopython package (62).

### 3D modeling

DNA model (B-form, 10.7 bp/turn, bend angle 210°) was constructed for 43 bp fragment of the consensus sequence (–63:–21 or 24:66 regions within periodic areas) using 3D-DART web service (63). The model was manually docked with the structure of *E. coli* DNA gyrase CTD (1zi0 (64)) in PyMOL (PyMOL Molecular Graphics System, Version 1.8 Schrödinger, LLC).

### Rif Topo-Seq and Rif Topo-qPCR with transcription depressed cells

Topo-Seq and Topo-qPCR experiments were performed as described above with the only exception that cells were pretreated with RNA-polymerase inhibitor rifampicin (122 μM) for 15 min to stop transcription (65) before addition of DNA gyrase poison (10 μM ciprofloxacin).

### Estimation of *E. coli* genes transcription level

3′-end RNA-Seq data was taken from publically available dataset (GEO dataset GSE95567) for *E. coli* K-12 DH10B cultivated under similar conditions (rich medium, 37°C, grown until OD_600_ ∼0.3) (66). Transcription level of a particular gene was calculated as an average sequencing depth of this gene.

### Determination of chromosomal macrodomains boundaries in *E. coli* W3110 Mu SGS

Macrodomains determined for *E. coli* MG1655 by Valens *et al.* (67) were applied to *E. coli* W3110 Mu SGS with modifications caused by Ori region inversion in W3110 strain in comparison to MG1655 as discussed in Duigou and Boccard (68). Differences between macrodomains of the two strains summarized in Supplementary Table DS2.

### DNA gyrase *in vitro* assays

133 bp DNA fragments (Supplementary Table S2) for *in vitro* experiments were ordered in GenScript as pUC19 clones (HincII site). Plasmids were routinely obtained from *E. coli* NEB5α (New England Biolabs) and purified using GeneJET Plasmid Miniprep Kit (Thermo Scientific). For EMSA and competition assays DNA fragments were PCR amplified using pUC19_for and pUC19_rev primers (Supplementary Table S2) and purified with Gel Extraction and DNA Cleanup Micro Kit (Thermo Scientific).

For electrophoretic mobility shift assay (EMSA) 25 nM of DNA fragments (Mu SGS, Consensus sequence or Scrambled consensus) were mixed with different amounts of reconstituted gyrase (0, 1, 2.5, 5 or 10× excess) holoenzyme (GyrA_2_GyrB_2_), incubated for 30 min at 25°C and run on 5% polyacrylamide gel in TBM buffer (90 mM Tris-borate, pH 7.5, 4 mM MgCl_2_). After the run gels were stained with SYBR Gold for 20 min and visualized under UV light.

For ATP-independent relaxation assay purified plasmids (∼9.5 nM) were mixed with gyrase holoenzyme (GyrA/GyrB, ∼7.5 nM) in a reaction buffer (35 mM Tris–HCl pH 7.5, 24 mM KCl, 4 mM MgCl_2_, 0.1 mg/ml BSA, 6.5% glycerol, 2 mM DTT) and incubated at 37°C for indicated times (0, 30, 60 min). Reactions were stopped with 1 volume of chloroform:isoamyl alcohol 24:1 and 1 volume of STEB (10 mM EDTA, 40% sucrose, 0.5 mg/ml bromophenol blue, 100 mM Tris–HCl pH 7.5). Aqueous layer was collected and topoisomers were separated in 1% TAE agarose gel with subsequent ethidium bromide staining.

For supercoiling, ∼9.5 nM of relaxed plasmids were combined with DNA gyrase holoenzyme (∼2.3 nM) in a reaction buffer (35 mM Tris–HCl pH 7.5, 24 mM KCl, 4 mM MgCl_2_, 0.1 mg/ml BSA, 6.5% glycerol, 2 mM DTT, 1.8 mM spermidine, 1 mM ATP) and incubated at 37°C for indicated times (0, 30, 60 min). Reactions were stopped and products were analyzed as described for ATP-independent relaxation assay. Supercoiling assays in the presence of competitor were done as above but 4.5 nM gyrase holoenzyme was incubated with ∼6 nM relaxed pBR322 substrate (Inspiralis) for 30 min at 37°C in the presence of required amounts of linear competitor.

For ATP-dependent relaxation, 80 nM of GyrA59_2_GyrB_2_ was mixed with 9.3 nM of purified plasmids in a reaction buffer (35 mM Tris–HCl pH 7.5, 125 mM KCl, 8 mM MgCl_2_, 0.36 mg/ml BSA, 6% glycerol, 5 mM DTT, 1.4 mM ATP) and incubated at 37°C for indicated times (0, 30, 60 min). Reactions were stopped and products were analyzed as described for ATP-independent relaxation assay.

### Quantification and statistical analysis

During GCSs-calling procedure Audic and Claverie test (59) was used to estimate enrichment significance with *P*-value <0.0025 as a threshold (custom Python script). Overrepresentation of GCSs in macrodomains, BIME-2 elements and downstream regions of rRNA operons was shown using binomial test (Supplementary Tables DS2, DS3, and DS7A). Statistical analysis of the number of GCSs in upstream, beginning, end, and downstream regions of genes and operons depending on their transcription status also based on binomial test (Supplementary Figure DS8, Supplementary Figure S13). Binomial test was used to estimate the number of GCSs that colocalize with top-scored genome sites (Supplementary Table DS4B). Binomial test with *P*-value cutoff = 0.001 was used to estimate statistically fluctuations in the numbers of GCSs over the *E. coli* genome (Figure 3, Supplementary Figure S11, custom python script). Fisher exact test was used to compare the numbers of GCSs in upstream, body, and downstream regions of rRNA operons between Cfx and RifCfx conditions (Figure 6C, custom Python script). To test the enrichment in the number of GCSs that fall into BIME-2 and which increase their N3E values after treatment with rifampicin, and to test the enrichment in the number of GCSs downstream of rRNA operons which decrease N3Es after rifampicin treatment, we used binomial test (Figure 6D, custom Python script). To identify significant bias in nucleotide frequencies during gyrase motif construction we used binomial test (Supplementary Figure S4, Supplementary Figure S5A, custom Python script). To identify associations of GCSs with TADs and sites of spontaneous mutations binomial test was used (Supplementary Table DS8, DS9, custom python script). Topo-qPCR experiments were performed in triplicate (Supplementary Table DS1), enrichment comparison was performed with t-test (Figure 6A, custom R script). T-test was used to compare score for BIME-2 regions, H-NS occupied areas, and downstream regions of different TUs sets (Supplementary Table DS7B) with *E. coli* genome score (custom python script). *t*-test was used to estimate deviation of score values of GCSs sets associated with TUs downstream regions (Supplementary Table DS7B). Exact conditional test for Poisson-distributed values (69) was used to estimate the deviation of N3E values for GCSs sets associated with TUs downstream regions (Supplementary Table DS7B). Fisher exact test and Chi-square test were used to estimate the significance of overlap between MatP and MukB binding sites (Supplementary Table DS6A).

## RESULTS

### Topo-Seq allows precise localization of gyrase cleavage sites

To efficiently sequence purified DNA fragments with covalently linked gyrase peptides, we applied a single-strand paired-end sequencing protocol (see Methods) to get rid of modified DNA chains at the library preparation step (Supplementary Figure S1). Thus, only free chains with 3′-ends directed towards the gyrase catalytic site are being sequenced, resulting in a specific structure of enrichment peaks at GCSs. Each of them should have a characteristic bimodal shape with a sharp 4-bp gap in the middle. Sequences to the left of the gap should align in a forward orientation with the GCS; their 5′-ends should vary, while 3′-ends should be identical and form the left ‘wall’ of the central 4-bp gap. Sequences to the right of the gap should similarly align in reverse orientation.

**Figure 2.**
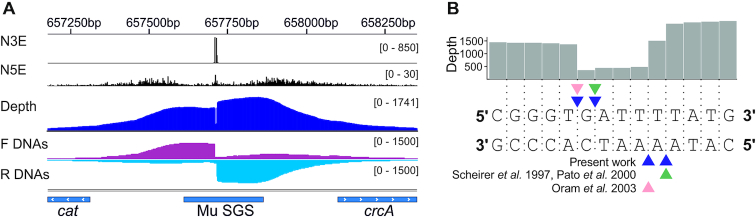
Signal structure at the strong gyrase binding site from bacteriophage Mu (Mu SGS). (**A**) Profiles of the number of 5′- and 3′-ends (N5E and N3E correspondingly) are shown in black. Total coverage depth (Depth) is in blue and coverages for DNA fragments that were aligned in forward and reverse orientations (F DNAs and R DNAs correspondingly) are in red and sky blue respectively. Tracks height (depth) are shown in brackets. The data visualized in IGV (58). (**B**) Close-up of the cleavage site. Coverage depth around the site is shown as a grey track, local sequence lies below. Cleavage sites known from the existing literature are shown (31,45,46).

To validate Topo-Seq, we constructed an *E. coli* strain with the Mu SGS inserted into a non-essential region of the genome. Exponentially growing cells were treated with several DNA gyrase inhibitors - ciprofloxacin (Cfx), microcin B17 (Micro), and oxolinic acid (Oxo) and subjected to Topo-Seq. While a weak signal was observed in control untreated cells at Mu SGS, in the presence of the inhibitors there was a dramatic increase in the abundance of intermediate complexes, resulting in a strong signal (Figure 2A). Concentrations of poisons used in Topo-Seq were chosen to be well above experimentally determined minimal inhibitory concentrations (MICs) for our strains (Supplementary Table S1). The shape of a signal at Mu SGS fully matched the expectations based on gyrase catalytic mechanism and the sequencing protocol used. The positions of the 4-bp gap ‘walls’ coincided with cleavage positions observed in previous biochemical studies of gyrase complexes trapped on Mu SGS *in vitro* (31,45,46) (Figure 2B). Thus, our procedure allows for accurate single-nucleotide identification of GCSs *in vivo*.

### Thousands of DNA gyrase cleavage sites are distributed throughout the *E. coli* genome

We next investigated the global distribution of GCSs in cells treated with gyrase poisons. We used the hallmark 4-bp gaps between 3′-ends of Topo-Seq enriched DNA fragments to develop an automatic GCS-calling procedure. Plotting the number of 3′-ends (N3E) allowed us to globally identify pairs of enriched positions (gap walls) separated by 4-bp gaps. We interpret these signals as gyrase trapping sites; the heights of gap walls provide an estimate of the relative number of gyrase binding events that initiate the strand passage step at a particular site.

**Figure 3.**
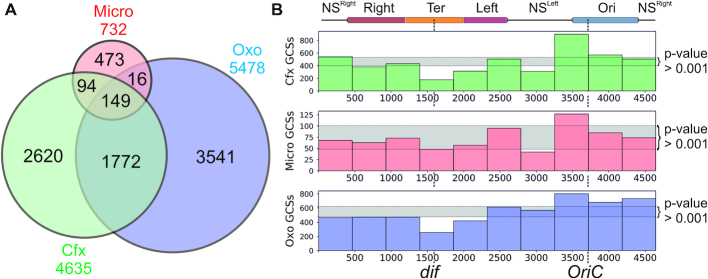
Comparison of GCSs sets observed with Cfx, Micro, and Oxo. (**A**) Venn diagram representation of the relations between GCSs sets obtained with different gyrase poisons. (**B**) General distribution of poison-mediated GCSs revealed by Topo-Seq. *E. coli* W3110 MuSGS genome is divided into 10 non non-overlapping bins, the height of the bars indicates the number of GCSs in a particular genome region. Area between 0.0005 and 0.9995 quantile values (distribution of the number of GCSs in a bin set as binomial) are shown in light gray, so bars that fall out of this zone have a statistically significant deviation from a value expected for the uniform distribution. NS^Right^—right non-structured region; Right—right macrodomain; Ter—terminator domain; Left—left macrodomain; NS^Left^ – left non-structured region; Ori—origin domain. Macrodomains set as in (68).

GCSs were detected as significantly enriched signals (statistical test from (59), *P*-value < 0.0025) during the two-step normalization procedure (see STAR Methods) and have passed additional filtering as being shared between at least two out of three biological replicas that were made for each gyrase inhibitor. In total, 4635 GCSs distributed throughout the genome were identified in Cfx-treated cells, 5478 in Oxo-treated, and 732 in Micro-treated cells (Supplementary Table S1). 41% of GCSs identified in the presence of Cfx are shared with Oxo set. The level of GCSs common for Micro and Cfx, or Micro and Oxo-treated cells is lower (33% and 23% of Micro GCSs, respectively), consistent with higher degree of similarity between Cfx and Oxo compared to a non-quinolone poison (Figure 3A). We found that GCSs revealed simultaneously by several drugs tend to have stronger signals (Supplementary Figure S17). We also found that the number of GCSs depends on the concentration of the poison used: for example, using 30–50-fold excess of Cfx over MIC we got 50–250 GCSs, while 300–600-fold excess gave 6000–7000 GCSs. Similarly, a 5-fold increase in Micro concentration resulted in the rise of the number of GCSs from 500–700 up to more than 3000 (Supplementary Table S1). Thus, the relatively low number of GCSs observed with Micro (732 GCSs at 30–40× MIC) is likely due to lack of saturation. Interestingly, compared to Oxo, Cfx is a much more effective cell growth inhibitor. Yet, judging by number of GCSs revealed by Topo-Seq, 120 μM Oxo (120–180× MIC) traps gyrase significantly more effectively than 10 μM Cfx (300–600× MIC). These observations seem to suggest that gyrase capture and subsequent cell death may be mediated by different processes. Indeed, differences in killing mechanisms between oxolinic acid and newer generations of quinolones have been reported (70).

A comparison of GCSs detected in strains with or without Mu SGS revealed no noticeable differences (apart from the expected strong gyrase enrichment at Mu SGS in the former strain) indicating that insertion of Mu SGS does not significantly affect genome-wide gyrase distribution (Supplementary Figure S2).

When regions flanking Mu SGS and another strong GCS (site downstream of rRNA A operon) identified by our procedure were tested by Topo-qPCR (for Cfx), enrichment was detected, and extent of this enrichment corresponded to levels of Topo-Seq signals. In contrast, no enrichment was observed during qPCR with primers specific for control sites where no gyrase cleavage was detected by Topo-Seq (Pearson's correlation: 0.94) (Supplementary Figure S3, Supplementary Table DS1). We conclude that our peak calling procedure is robust and reflects the *in vivo* positioning of gyrase intermediates trapped by the inhibitor.

The analysis of the *E. coli* chromosome architecture (67,68) revealed the presence of large topologically independent macrodomains located around the replication origin and terminator region (Ori and Ter macrodomains, respectively). Ter is flanked with Left and Right macrodomains, separated from Ori by the left and right non-structured regions of the chromosome (NS^left^ and NS^right^, correspondingly). We observed statistically significant overrepresentation of GCSs in NS^left^ and Ori and underrepresentation in Ter and Left (binomial test, *P*-values are <1e-3) (Figure 3B and Supplementary Table DS2, Supplementary Figure S11). This pattern could not have been due to intensive DNA replication in actively dividing cells since the two-step normalization procedure used for GCSs calling involved normalization for local copy number/sequencing depth.

Topo-Seq data was compared with available ChIP-chip gyrase binding data (24). Despite the differences between the two approaches and the resolution of final data, we found a significant positive correlation between both datasets and a very similar distribution of signals over the *E. coli* genome (Supplementary Figure S15). Thus, gyrase binding and cleavage are likely to be connected, additionally validating the Topo-Seq methodology.

### DNA gyrase has an extensive and degenerate binding motif

**Figure 4.**
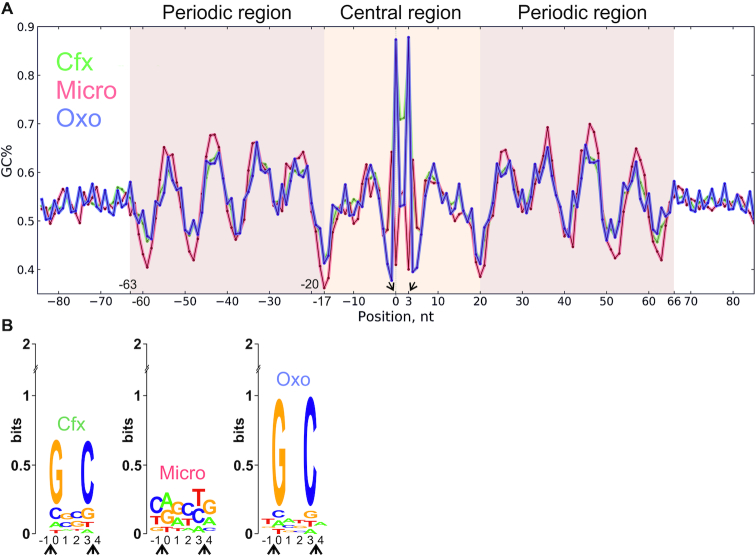
DNA gyrase has a binding motif revealed with a range of poisons: Cfx, Micro, and Oxo. (**A**) DNA sequences under GCSs were extracted and aligned; resulting motif, shown as a plot of GC content, has a central region (–16:19 nt) around the cleavage site, and two periodic regions (–63:–17 and 20:66 nt). (**B**) Logo representation of motif's central part around cleavage site. In the coordinates chosen, DNA gyrase cleaves forward chain between -1 and 0 and reverse chain between 3 and 4 bp (cleavage events are indicated with arrows).

Single-nucleotide resolution GCS mapping allowed us to directly look for potential sequence preferences of the gyrase in antibiotic-treated cells. Aligning of sequences flanking the positions of GCSs revealed a significant deviation from random frequencies of nucleotides indicating the presence of a potential motif. When the obtained frequency matrix was converted to GC% (see Materials and Methods for details), similar 130-bp degenerate pattern was observed for signals obtained from cells treated with each of the gyrase poisons tested (Figure 4A, Supplementary Figure S4). The pattern is symmetrical with respect to the cleavage sites, which are located between –1/0 and +3/+4 positions of the motif. We refer to this pattern as the ‘gyrase motif’. It consists of a central (from –16 to 19 bp) part containing the cleavage site and two flanking (from –63 to –17 bp and from 20 to 66 bp) regions, each with periodic (10.75 bp) changes in GC% resembling the binding pattern of eukaryotic nucleosomes (71) (see Supplementary Figure S5). Analysis of calculated DNA geometry for GCSs sequences using GBshape database (72) revealed noticeable deviations for such parameters as helix twist, propeller twist, and minor groove width (Supplementary Figure S14). This observation may indicate that DNA recognition by the enzyme occurs through indirect readout (73).

The only significant differences between motifs obtained with different inhibitors were observed at the cleavage site (Figure 4B). GCS motifs obtained with Cfx and Oxo-treated cells confirmed the well-established tendency of quinolones to intercalate and facilitate scission before guanine residues (34,74,75). In contrast, this pattern was not observed in the case of Micro, which likely has a different mode of interaction with gyrase and/or DNA (76), leading to a different consensus in the central part of GCS. This difference in cleavage preferences may explain a poor overlap between the Micro and quinolones GCSs sets (Figure 3A). When sequences under the GCSs were screened for overrepresentation of known motifs with Tomtom (77), none were detected, suggesting that gyrase trapping is independent of other DNA binding proteins.

### Gyrase activity correlates with sequence properties

To remove antibiotic-specific biases from the gyrase motif, the frequency values for central positions most affected by antibiotics (0–3 bp) were made equal to baseline nucleotide frequencies of the *E. coli* genome (resulting position frequency matrix and consensus sequence can be found in Supplementary Table DS10). The resulting position-weight matrix corresponding to a ‘combined’ gyrase motif was used to scan the genome of bacteriophage Mu and the pBR322 and pSC101 plasmids. As expected, previously known strong gyrase sites from Mu, pSC101 and pBR322 (33,34,46) were identified as having the highest scores (Supplementary Table DS4A). When the genome of *E. coli* DY330 Mu SGS was scanned, Mu SGS had the third highest score. Five out of 13 highest scoring *E. coli* sites were among the GCSs identified by Topo-Seq, a highly significant (binomial test, *P*-value: 8.4e–14) overrepresentation. In addition, for 8 out of 13 highest scoring sites, there was a total of 27 GCSs within 5 bp of their central regions (Supplementary Table DS4B) providing independent support for the relevance of the motif (binomial test, *P*-value: ∼0) and suggesting some flexibility of the cleavage sites within the motif.

Analyzing the predictive power of gyrase motif score, we found that GCSs located in sequences with higher scores tend to have higher N3E values (Supplementary Figure S6), an evidence that gyrase activity depends on DNA substrate sequence composition. Correlations between N3E and score are small, but statistically significant for all Topo-Seq experiments (Pearson's correlation: 0.22, 0.15 and 0.23 for Cfx, Micro and Oxo respectively with *P*-values < 4.3e–5). N3E and score values for GCSs revealed by more than one poison are higher (Supplementary Figure S17) meaning that 149 GCSs reproduced in experiments with all three drugs might be the most preferred gyrase sites in the genome.

### The DNA gyrase consensus sequence strongly binds the enzyme and inhibits its activity *in vitro*

**Figure 5.**
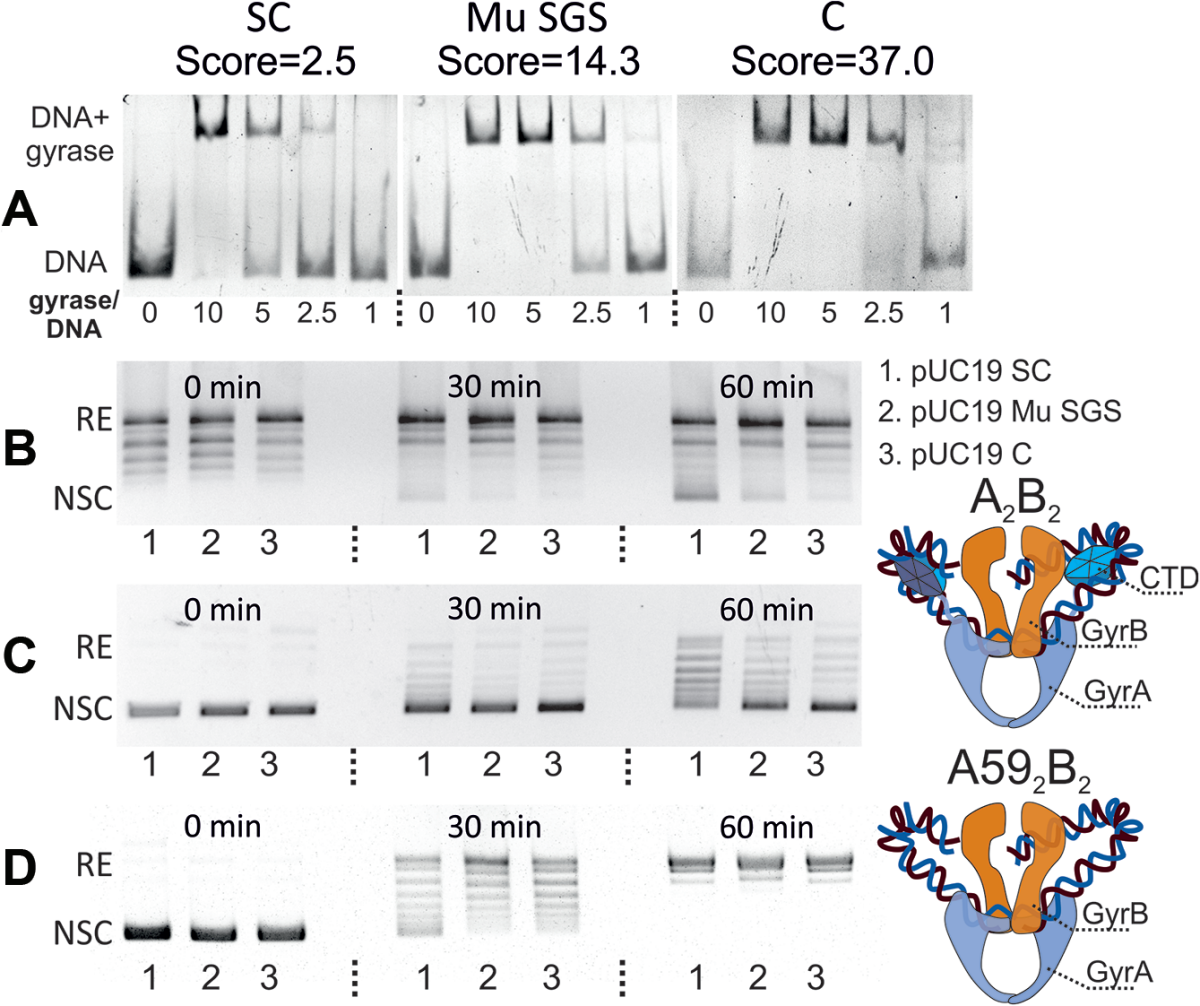
DNA gyrase behavior depends on a DNA substrate gyrase binding score. (**A**) EMSA analysis of DNA gyrase binding with 133 bp fragments; fragments scores are showed above the pictures; molar gyrase/DNA ratio is indicated under the lanes. SC—scrambled consensus, C—consensus. (**B**) Time-course of gyrase supercoiling of pUC19 plasmids harboring indicated 133 bp fragments; RE—relaxed plasmid, NSC—negatively supercoiled plasmid. 1—scrambled consensus, 2—Mu SGS, 3—consensus. (**C**) Time-course of ATP-independent relaxation of pUC19 plasmids harbouring indicated 133 bp fragments. (**D**) Time-course of ATP-dependent relaxation of the same plasmids by truncated GyrA59_2_GyrB_2_ gyrase lacking CTD.

To test the observations that specific properties of DNA sequence expressed in the terms of score can explain elevated gyrase activity (estimated as a number of GCSs or N3E value) at a particular locus, we performed *in vitro* experiments with a set of 133 bp synthetic DNA sequences having different degree of similarity to the gyrase binding motif: a consensus sequence (calculated score 37), Mu SGS (calculated score = 14.3) and scrambled consensus (score = 2.5) (Supplementary Table S4). As can be seen from Figure 5A, affinities of DNA fragments for gyrase were roughly proportional to their scores in the EMSA assay. Linear DNA fragments encompassing Mu SGS and consensus sequence but not scrambled consensus, inhibited supercoiling of pBR322 plasmid by DNA gyrase when added as competitor DNA (Supplementary Figure S12). To more directly look at gyrase activity, we investigated ATP-dependent supercoiling and ATP-independent relaxation of pUC19-based plasmids carrying cloned fragments described above by DNA gyrase. Surprisingly, the supercoiling and relaxation efficiency of plasmids was inversely related to cloned insert score (Figure 5B and C): DNA gyrase processed substrates carrying consensus sequence significantly slower than substrates with scrambled consensus or even Mu SGS. In contrast, ATP-dependent relaxation of plasmids by GyrA59/GyrB gyrase mutant lacking CTD proceeded at comparable rates (Figure 5D). Taking into account that CTDs are crucial for DNA wrapping around the enzyme and supercoiling (78,79) we propose that CTDs recognize the phased bending signals in the ‘arms’ of the motif and that strong interactions between DNA and CTDs may inhibit structural transitions required for the catalytic reaction.

### Gyrase is attracted to the regions downstream of transcribed loci

Based on the ‘twin-domain model’ and the fact that gyrase prefers positively supercoiled DNA to act upon, we expected the enzyme to be preferentially found downstream of transcribing RNAP (17,20). Whole-genome analysis revealed that in general less GCSs associate with poorly transcribed transcription units (TUs) than with highly transcribed ones (binomial test, *P*-value ∼0), as has been also observed in *M. tuberculosis* (23). We have screened the highly transcribed loci for their association with GCSs and found that cleavage sites significantly accumulate in extended downstream regions of active TUs (Supplementary Figure S8, Supplementary Figure S13). The enrichment is most noticeable for rRNA operons, which have the highest transcription rate (80) (Supplementary Table DS7A).

**Figure 6.**
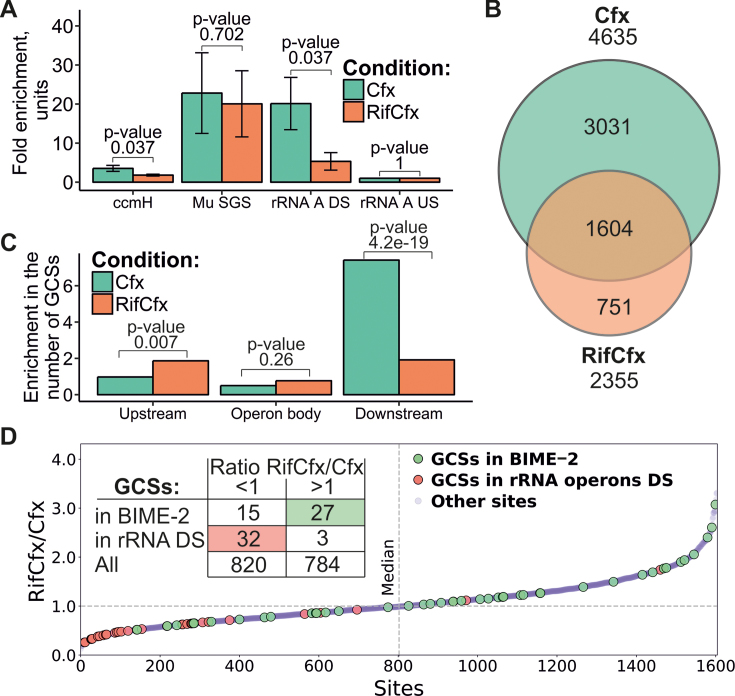
Transcription facilitates gyrase activity. (**A**) Enrichment observed at several genomic sites by Topo-qPCR for Cfx and RifCfx (data for three replicas). Error bars constructed as ±2 standard errors, *P*-values for t-test for means are indicated above the bar pairs. (**B**) Venn diagram showing overlap of Cfx and RifCfx GCSs sets. (**C**) GCSs relocation from downstream regions of rRNA operons when transcription is inhibited with rifampicin. Enrichment in the number of GCSs, which is a ratio of the number of observed GCSs to the number of expected GCSs (statistic—Fisher exact test), is plotted. (**D**) GCSs shared between Cfx and RifCfx sorted by ratio of their N3E values. GCSs that fall in 5 kb downstream regions of rRNA operons (red dots) have a significant tendency to have lower N3E values when cells are treated with Rif (binomial test, *P*-value = 0.015). On the other hand, GCSs that fall into REPs (green dots) reveal tendency to increase N3E under the same conditions (binomial test, *P*-value = 4e–7).

To experimentally test whether transcription affects the gyrase distribution, we treated cells with RNAP inhibitor rifampicin (Rif) before Cfx-mediated gyrase trapping (the procedure further referred as RifCfx). While the overall number of cleavage sites dropped twofold after Rif treatment (4635 for Cfx versus 2355 for RifCfx, Figure 6B) their average strength (measured as N3E value) and sequence specificity (measured as gyrase binding score value) slightly increased (Supplementary Figure S7). Rifampicin did not affect either overall shape of gyrase motif, or local cleavage properties characteristic of Cfx (Supplementary Figure S9). At the same time, the global gyrase gradient became altered and less pronounced (Supplementary Figure S11B). Most notably, Rif eliminated both the accumulation of GCSs in the downstream regions of rRNA operons (Figure 6C) and avoidance of poorly transcribed TUs (Supplementary Figure S8B). The GCSs which remained downstream of rRNA operons decreased their signals (Figure 6A and D). In contrast, signals from some other sites, potentially not directly related to transcription intensity, for example GCSs located in BIME-2 regions, were increased in the presence of Rif (Figure 6D). Overall, we conclude that transcription inhibition with rifampicin leads to significant relocation of active gyrase, suggesting that transcription is a strong factor that attracts gyrase to downstream DNA.

### GCSs are overrepresented in a subset of BIME-2 sequences

We found significant overrepresentation of GCSs in BIME-2 elements in full agreement with previous experiments (36) (binomial test, *P*-value ∼0 for Cfx, 3e–4 for Micro, and 1e–9 for Oxo). In particular, BIME-2 located between *sucC* and *sucB* and between *tldD* and *yhdP* genes house multiple GCSs (Supplementary Table DS3). Closer look revealed that gyrase much more frequently cleaves y-type than z2-type repetitive extragenic palindromes (REPs) that form BIME-2 in consistence with old observations (35) and especially prefers loops and non-complementary regions within the stems of cruciform-forming REPs (Supplementary Figure S10). The number of GCSs-containing REPs within particular BIME-2 elements considered by Espeli and Boccard (36) is also similar to the previously observed number of gyrase-generated cuts. Thus, DNA gyrase binds at least some BIME-2s *in vivo*. BIME-2 locate in intergenic regions and accumulation of GCSs in them could be due to positive supercoiling associated with transcription of adjacent genes. In this case one would expect the gyrase signal to decrease when transcription is inhibited by Rif: however, the opposite is observed (Figure 6D). Interestingly, BIME-2 have a higher mean score than expected (–1.3 versus –2.3 over the *E. coli* genome; *t*-test, *P*-value ∼0), which may be a reason of their preferential binding by gyrase. Thus, BIME-2 have properties of transcription-independent strong gyrase sites and might contribute to genome organization.

### GCSs colocalize with MukB and avoid H-NS binding regions

Nucleoid-associated proteins (NAPs) contribute to genome organization and local topology. High-resolution maps of the binding sites of *E. coli* NAPs Fis, IHF, H-NS, MatP, and MukB are available (81–83). Genome-wide cleavage data are also published for *E. coli* Topo IV, which is a close paralog of DNA gyrase (49). We compared available datasets with our Topo-Seq data to find potential associations between gyrase activity and these proteins. We found that GCSs are slightly underrepresented at IHF sites and at Fis sites in Cfx (±Rif) and Oxo treated cells (binomial test, *P*-value < 0.002) but not in Micro-treated cells. GCSs are strongly underrepresented at H-NS binding regions (binomial test, *P*-value < 9.75e–23) for all experimental conditions (Supplementary Table DS6). H-NS, a well-known transcription repressor, primarily associates with silent regions of the genome (Supplementary Figure S16D and (82)). Hence, H-NS occupied areas are expected to lack transcription-mediated positive supercoiling. Sequences of H-NS occupied areas also score lower than the genome mean for gyrase motif (–4.4 versus –2.3, *t*-test *P*-value ∼0). A combination of these two factors may jointly contribute to gyrase avoidance.

Due to similarities in the structure and mechanism of Topo IV and gyrase, one could expect a correspondence between their cleavage sites distribution. However, our analysis did not reveal any significant association, either positive or negative, between GCSs and Topo IV cleavage sites (Supplementary Table DS6). Topo IV activity and distribution within a cell are thought to be positively connected to MukBEF—a complex involved in structural maintenance of chromosomes (9). In turn, MukBEF is known to physically interact with the MatP protein (83). MatP binds with high affinity to specific *matS* sites that concentrate in the Ter macrodomain (84), and displaces the MukBEF complex (83,85). Together, the Topo IV-MukBEF-MatP system is thought to coordinate the proper timing of replicating chromosome unlinking and segregation (83). We found that GCSs are overrepresented at MatP-occupied regions (binomial test, *P*-value < 0.006) but not in the immediate vicinity of *matS* sites (Supplementary Table DS6). Strikingly, the enrichment is even more pronounced for MukB sites (binomial test, *P*-value < 3.9e–14) (Supplementary Table DS6). In fact, the genome-wide MukB distribution is similar to that of the gyrase (Supplementary Figure S11) and MukB accumulates at extended regions downstream of TUs in transcription-dependent manner (Supplementary Figure S16C, Supplementary Figure S13C). Moreover, the gyrase binding motif score of MukB binding regions is relatively high (–1.5 versus –2.3 genome average, *t*-test *P*-value ≈ 0). Overall, we suggest that DNA gyrase likely acts independently of the MatP-MukBEF-TopoIV decatenation ensemble but the MukBEF complex might synergize with the gyrase downstream of active TUs by stabilizing plectonemes induced by transcription-dependent positive supercoiling.

### Lack of association between GCSs density, topologically associated domains (TADs), and sites of spontaneous mutations

DNA-gyrase supercoiling activity might contribute to bacterial genome structuring (28,86). We therefore looked for association of GCSs with TADs, as defined for *E. coli* MG1655 (85). However, no significant over- or underrepresentation of GCSs were found either in TADs, the inter-TADs, or nearby the TADs borders (Supplementary Table DS8). Gyrase sites on DNA can also be expected to have an increased rate of spontaneous mutations due to association with double-strand breaks introduced by gyrase. We compared the distribution of spontaneous mutations throughout the *E. coli* genome (for wild-type and mutagenic *mutL* cells (87)) and found no pronounced associations between mutations and GCSs (Supplementary Table DS9).

## DISCUSSION

In this paper, we used Topo-Seq (drug-induced trapping and purification of covalent topoisomerase-DNA complexes, followed by application of a single-strand paired-end sequencing protocol) to robustly identify thousands of GCSs across the genome of *E. coli* treated with gyrase poisons. The results provide unprecedented, single-nucleotide precision view of gyrase localization throughout the chromosome. While our data relied on gyrase poisons to trap the enzyme on DNA, the similarities with ChIP study of gyrase binding (24) and commonality of genome-wide GCS patterns and binding motifs observed for unrelated inhibitors strongly suggest that our experiments provide information about the natural distribution of gyrase along the *E. coli* chromosome. This is further supported by the observation that the common GCS motif revealed by Topo-Seq strongly binds to the DNA gyrase *in vitro* in the absence of gyrase poisons.

We observed a global *ori-ter* gradient in the frequencies of GCSs which agrees well with ChIP-ChIP data obtained in the absence of poisons (24) (Supplementary Figure S15). The trend can be explained by either gyrase-binding score gradient (sequences near the origin have higher propensity to bind gyrase) or by transcription gradient (higher level of transcription near origin). When transcription is inhibited by Rif, the *ori-ter* gradient remains, although it becomes significantly less pronounced (Pearson's correlation between the number of GCSs and transcription level falls from 0.85 for Cfx dataset to 0.24 for RifCfx set). Thus, it seems that transcription primarily forms gyrase activity gradient over the genome by influencing local supercoiling state (Supplementary Figure S11). Gyrase activity might neutralize transcription-mediated positive supercoiling which leads to an even distribution of superheliсity over the genome in the phase of exponential growth as detected by psoralen binding assay (27).

**Figure 7.**
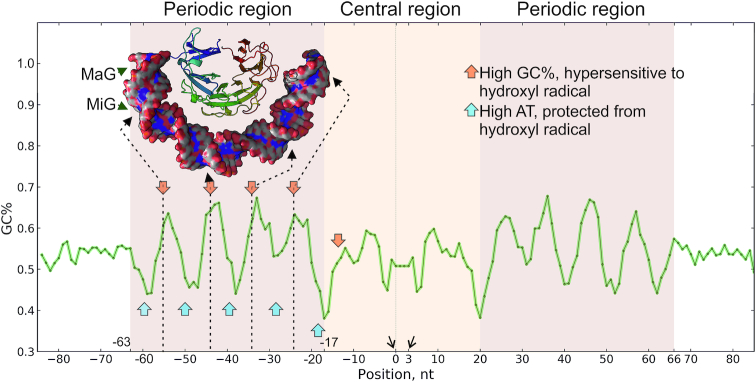
DNA gyrase binding motif reflects DNA wrapping around the CTDs. DNA gyrase ‘combined’ motif (based on datasets from all three different antibiotics tested) flanked with random regions shown in green. Orange arrows indicate GC-rich and hydroxyl radical hypersensitive sites, blue arrows—AT-rich and protected regions. The structure of DNA-wrapped CTD is shown above the motif, dashed arrows point hypersensitive sites in DNA’s minor groove. MaG—DNA major groove, MiG—DNA minor groove. Hydroxyl radical sensitivity data is taken from (91).

Identification of thousands of GCSs allowed us to delineate the gyrase binding motif characterized by phased variation in G/C content. This motif is too long (130 bp) and too degenerate to be identified by standard algorithms (MEME (88), ChIP-Munk (89), or Gibbs-sampler (90)). The 130-bp motif matches the length of DNA protected by the gyrase from hydroxyl radical cleavage (91). The central 36 bp part of the motif is contained within a 40–50 bp segment most strongly protected from DNase I cleavage in *in vitro* experiments, while the flanking 47-bp periodic regions are likely localized on the sides. The G/C content variation may be explained by DNA lying on protein surface in a way that its minor groove contains GC when open to solution, and AT when facing the protein. AT-rich tracts are known to be flexible (92) and tend to form bends when positioned in phase with DNA helix (93), as observed in the gyrase motif. The size of a periodic regions agrees well with the 35–52-bp minimal DNA fragments which are thought to be wrapped around GyrA CTDs (78) (Figure 7). Our *in vitro* experiments with DNA sequences having different levels of similarity to gyrase motif independently support this conclusion. Well-phased sequences (Mu SGS and consensus) bind to the gyrase much better than the scrambled one (Figure 5A) and cause the slowing down of enzyme (Figure 5BC). On the other hand, gyrase lacking CTDs shows no differences when relaxing different substrates (Figure 5D). That might mean that very strong binding detected for phased sequences could interfere with CTD movements crucial for optimal activity of the enzyme as discussed in (94,95). Interestingly, the same inhibition effect was previously observed for the pSC101 SGS (46), which we identified as a high-scoring sequence (score = 16), that might explain why there are no sequences in *E. coli* genome with a score higher than ∼15.

GCSs sets obtained with cells treated by different drugs overlapped only partially, which likely results from specific drug–DNA interactions in the central part of the motif at the cleavage site. Interestingly, Micro GCSs have weaker cleavage pattern compared to those obtained with quinolone drugs (Figure 4B). This may indicate that Micro does not intercalate into DNA with its oxazole/thiazole regions, and instead traps gyrase differently. This notion is consistent with an observation that the periodic part of Micro motif has the highest amplitude, possible reflecting periodic interaction of Micro oxazole/thiazole cycles with DNA bound to CTDs. Intriguingly, it's been shown previously that long DNA segments and strand passage are essential for Micro to form a stable cleavage complex (76).

Transcription-induced positive supercoiling is a well-known factor that attracts gyrase activity (21,23,24). Our data show that more DNA gyrase is associated with extended (∼2–5 kb) regions downstream of actively transcribed transcription units (Supplementary Figure S13). While such association is predicted by the Wang model, our results show that gyrase concentrates not directly in front of the elongating RNA polymerase but much further downstream of *in vivo* transcription termination sites (as determined in (96)), including areas of poorly transcribed downstream genes (Supplementary Figure S13D). Interestingly, attraction of gyrase to these downstream regions seems not to be hard-wired in the genome, since the gyrase motif scores of GCSs in downstream regions and scores of these regions themselves are the same or lower that throughout the rest of the genome (Supplementary Table DS 7B). The result underscores the driving role of transcription in populating these sites by the gyrase and explains the redistribution of gyrase observed upon transcription inhibition.

It appears that sequence properties and transcription are not the sole factors regulating gyrase activity. For example, some high-scoring sites are not GCSs and genome-wide correlation between gyrase binding score and N3E value does not rise significantly for Rif-treated cells, as could have been expected. No data are available for *E. coli* enzyme interactions with nucleoid structuring proteins, however local topology of nucleic acid modulated by them might influence gyrase behavior in a complicated way. Recently, for example, GapR protein was found in *Caulobacter crescentus*, that specifically interacts with positively supercoiled DNA and stimulates gyrase and Topo IV activity, possibly via interaction with them (97). To support this notion, we found statistically significant associations of gyrase sites with binding regions of several NAPs. While regions of H-NS and MukB binding shows strong associations with sites of gyrase activity (negative and positive respectively), link with other proteins is less pronounced. Negative correlation with IHF-occupied sites cannot be explained either by the distribution of the protein across the genome - like gyrase, IHF is more presented at Ori and less at Ter (Supplementary Figure S11H), or by association with silent genes - we did not find any transcription-dependent binding for IHF (Supplementary Figure S16B). Also, it is not based on sequence properties of IHF sites as their mean score is close to genome mean (–2.4 versus –2.3). Another weak negative association was found with Fis. It is known to bind in upstream regions of TUs (82) and we noticed that this effect is transcription-dependent (Supplementary Figure S16A). Score of Fis sites is just a little lower than genome mean (–2.7 versus –2.3) so this protein and gyrase might simply act on different sides of TUs. It should be noted, however, that Fis is a repressor of transcription of both the *gyrA* and *gyrB* genes in *E. coli* and *Salmonella* (98), while *fis* transcription itself is stimulated by high levels of negative supercoiling (99).

It is more difficult to explain a positive association between GCSs and MatP sites which exists only for non-Ter and at the same time weak (<3-fold enrichment) sites (Supplementary Table DS6). In contrast, all Ter sites show mainly negative association with GCSs, while strong (>3-fold MatP enrichment) and non-Ter regions lack any associations. We can explain this by binding between MukBEF and MatP, that results in a colocalization of binding sites (Fisher exact test, *P*-value = 6.27e–17) (Supplementary Table DS6A). This hypothesis still needs to be tested experimentally.

Global transcriptional responses to environmental factors can be mediated by supercoiling via gyrase or other topoisomerases (1,100,101). Use of trapping agents to catch the enzymes *in flagrante delicto* on a nucleic acid is a promising alternative for non-specific formaldehyde-mediated ChIP-Seq approaches (49,50). The combination of a specific sequencing protocol with a cleavage pattern detecting algorithm results in single-nucleotide precision of Topo-Seq. Our method can be directly applied to different bacteria including pathogens such as *Salmonella* or *Mycobacteria*, whose global supercoiling dynamics must be significantly different from that of *E. coli* (102,103), and to *Caulobacter crescentus* and genome-reduced *Mycoplasma*, in which supercoiling was shown to contribute to chromosomal domain organization (104,105). The Cfx-sensitive gyrase is found in eukaryotes such as plants and *Apicomplexa* (106–108). The yet-unknown function of the enzyme in these organisms can be assessed using Topo-Seq. Finally, our method can be easily modified to study a larger variety of enzymes (other topoisomerases, recombinases etc.) that form intermediate covalent complexes with nucleic acids in the presence of stabilizing poisons.

## DATA AVAILABILITY

All software used for data processing and analysis is freely available. Pipeline for raw sequencing data analysis and scripts for data visualization and statistical analysis: https://github.com/sutormin94/Gyrase_Topo-seq. Raw sequencing data: GEO: GSE117186. Genome-wide analysis of GCSs correlations with NAPs binding regions, TUs expression, TADs positions, BIMEs positions, mutations, etc.: https://github.com/sutormin94/Gyrase_Topo-seq//Additional_genome_features.

## Supplementary Material

Supplementary Data
