# Single-nucleotide-resolution mapping of DNA gyrase cleavage sites across the Escherichia coli genome

**DOI:** 10.1093/nar/gky1312

**Published:** 2019-01-09

**Authors:** Dmitry Sutormin, Natalia Rubanova, Maria Logacheva, Dmitry Ghilarov, Konstantin Severinov

**Affiliations:** 1 Centre for Life Sciences, Skolkovo Institute of Science and Technology, 143026 Moscow, Russia; 2 Department of Bioengineering and Bioinformatics, Lomonosov Moscow State University, 119991 Moscow, Russia; 3 Malopolska Centre of Biotechnology, Jagiellonian University, 30387 Cracow, Poland; 4 Waksman Institute for Microbiology, Rutgers, The State University of New Jersey, Piscataway, NJ 08854, USA

*Nucleic Acids Research*, 2018, https://doi.org/10.1093/nar/gky1222

The Authors apologize for an error in reference (98) which should be:
