# Supplementary material for: Single-nucleotide-resolution mapping of DNA gyrase cleavage sites across the Escherichia coli genome
Source: Nucleic Acids Res. 2018 Dec 4;47(3):1373–88. doi: 10.1093/nar/gky1222 (PMC6379681; doi:10.1093/nar/gky1222)
Supplement: Supplementary Data [file gky1222_supplemental_files.zip › Supplementary_Figures_191118.pdf]

# Single-nucleotide-resolution Mapping of DNA Gyrase Cleavage Sites Across the *Escherichia coli* Genome

**Dmitry Sutormin[1,2], Natalia Rubanova[1], Maria Logacheva[1],**

**Dmitry Ghilarov[1,3]* and Konstantin Severinov[1,4]***

[1]Centre for Data-Intensive Biomedicine and Biotechnology, Skolkovo Institute of Science and Technology, 143026 Moscow, Russia

[2]Department of Bioengineering and Bioinformatics, Lomonosov Moscow State University, 119991, Moscow, Russia

[3]Malopolska Centre for Biotechnology, Jagiellonian University, 30348, Cracow, Poland

[4]Waksman Institute for Microbiology, Rutgers, The State University of New Jersey, Piscataway, NJ 08854, USA

*Correspondence should be addressed to:

dmitry.gilyarov@uj.edu.pl

severik@waksman.rutgers.edu

§Author's current address

# SUPPLEMENTARY FIGURES

Supplementary Figure S1. **DNA gyrase mechanism of action and sequencing libraries preparation protocol allows to identify cleavage sites with single-nucleotide resolution**. (**A**) Coverage depth in the close vicinity of Mu SGS; cleavage site sequence is written under the depth track; known cleavage positions (with references) marked with colored triangles. (**B**) Schematic view of double strand DNA fragment cleaved with DNA-gyrase; 5'-ends blocked with peptides including catalytic $Tyr^{122}$ (Y) labeled with yellow. (**C**) dsDNA is melted giving 4 chains. Terminal Deoxynucleotidyl Transferase (TDT) adds Gs to the free 3'-ends; single strand Adapter 1 aligns to the oligoG region with its oligoC track; Klenow fragment fills the gap to make dsDNA. (**D**) Double strand Adapter 2 ligates with free blunt ends. Ligation is inefficient when the end is blocked with peptide (in yellow). (**E**) Sequencing starts from Adapter 2 (arrows). (**F**) Legend for adapters

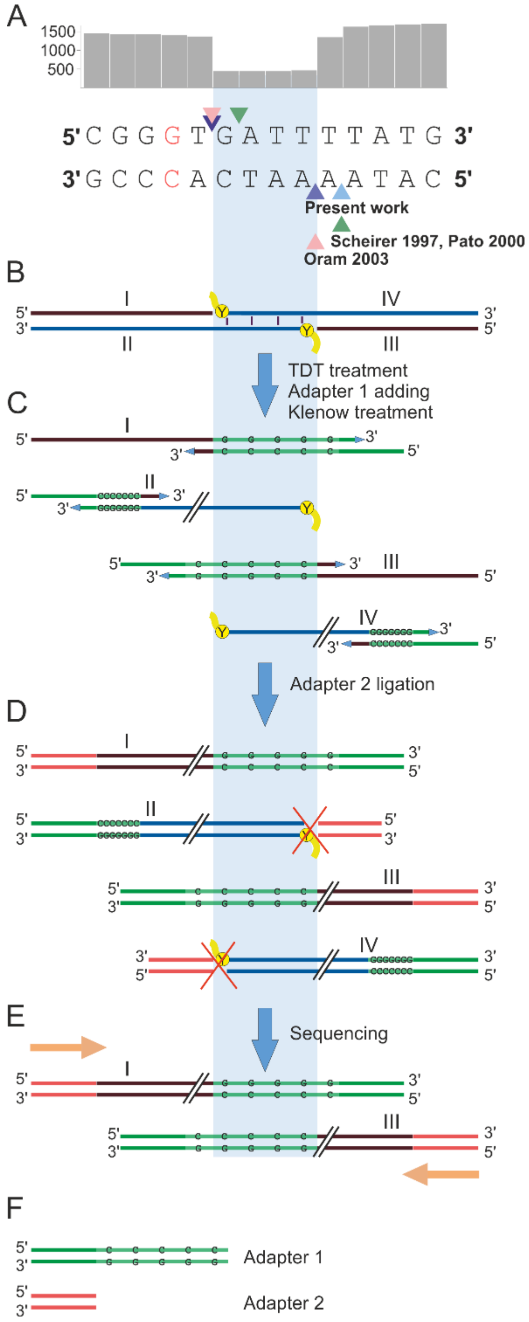

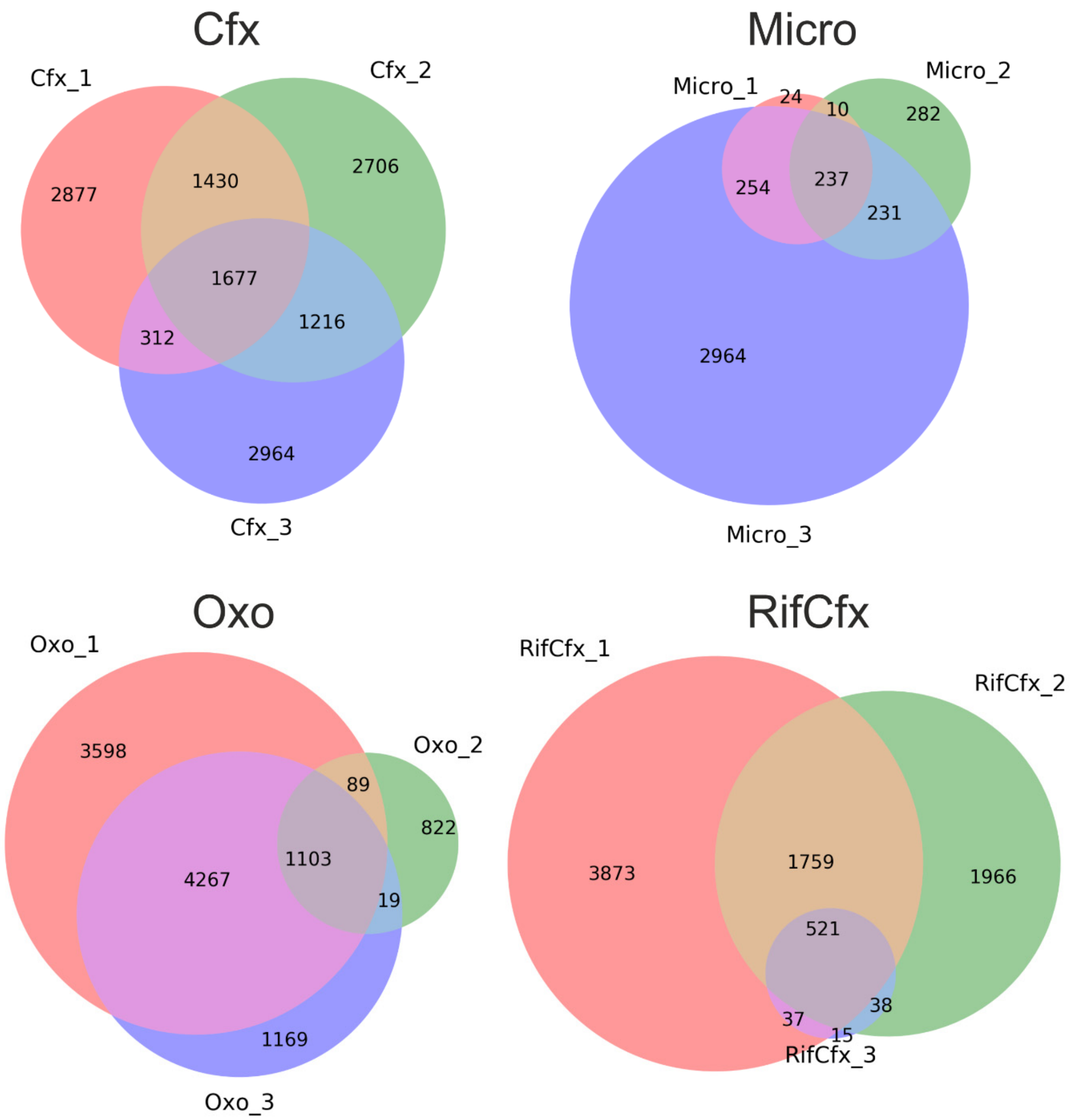


Supplementary Figure S2. **Agreement between GCSs sets obtained in different biological replicas of Topo-Seq experiments.** Relationships between GCSs sets are presented as a Venn diagram. For each condition replicas 1 and 2 were made with *E. coli* DY330 Mu SGS and replica 3 – with *E. coli* DY330.

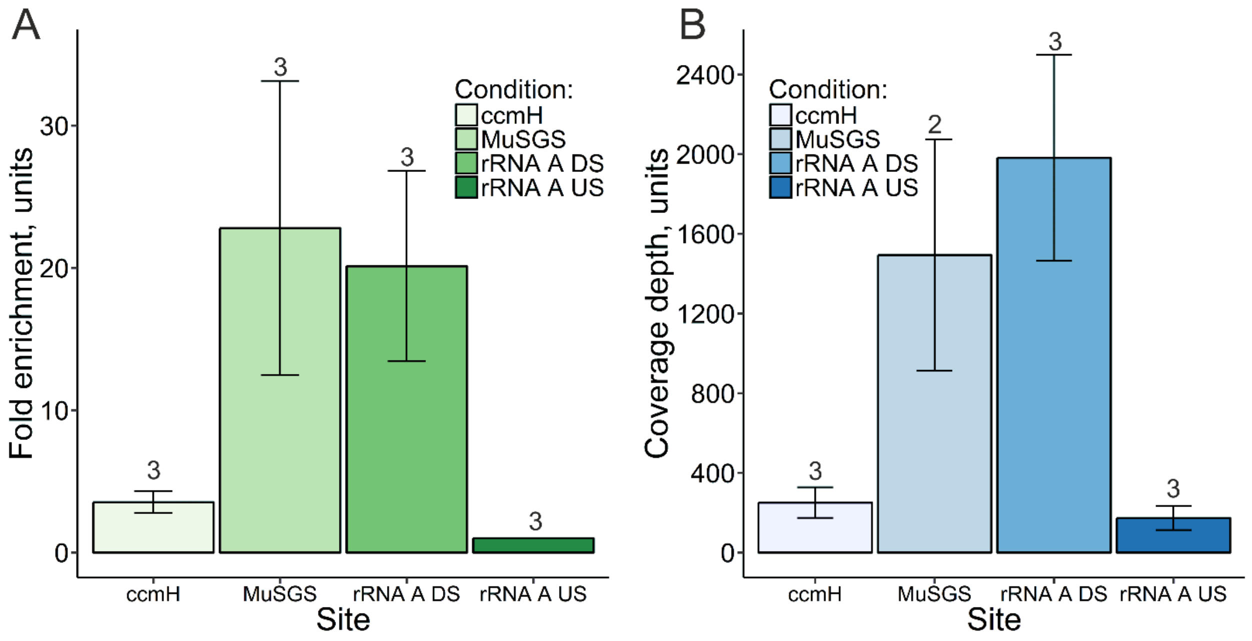


Supplementary Figure S3. **Comparison of the results of Cfx-mediated Topo-qPCR and Topo-Seq**. Enrichment was estimated for four sites: **Mu SGS**, **ccmH**, **rRNA A DS** and **rRNA A US** (short description in **Supplementary Table S2**). Numbers above the error bars indicate the amount of biological replicas, error bars constructed as +/- 2 standard errors. (**A**) Fold enrichment observed on genome sites indicated with Topo-qPCR. Raw cycle threshold (Ct) data is stored in **Supplementary Table DS1**. (**B**) Coverage depth observed on genome sites indicated with Topo-Seq.

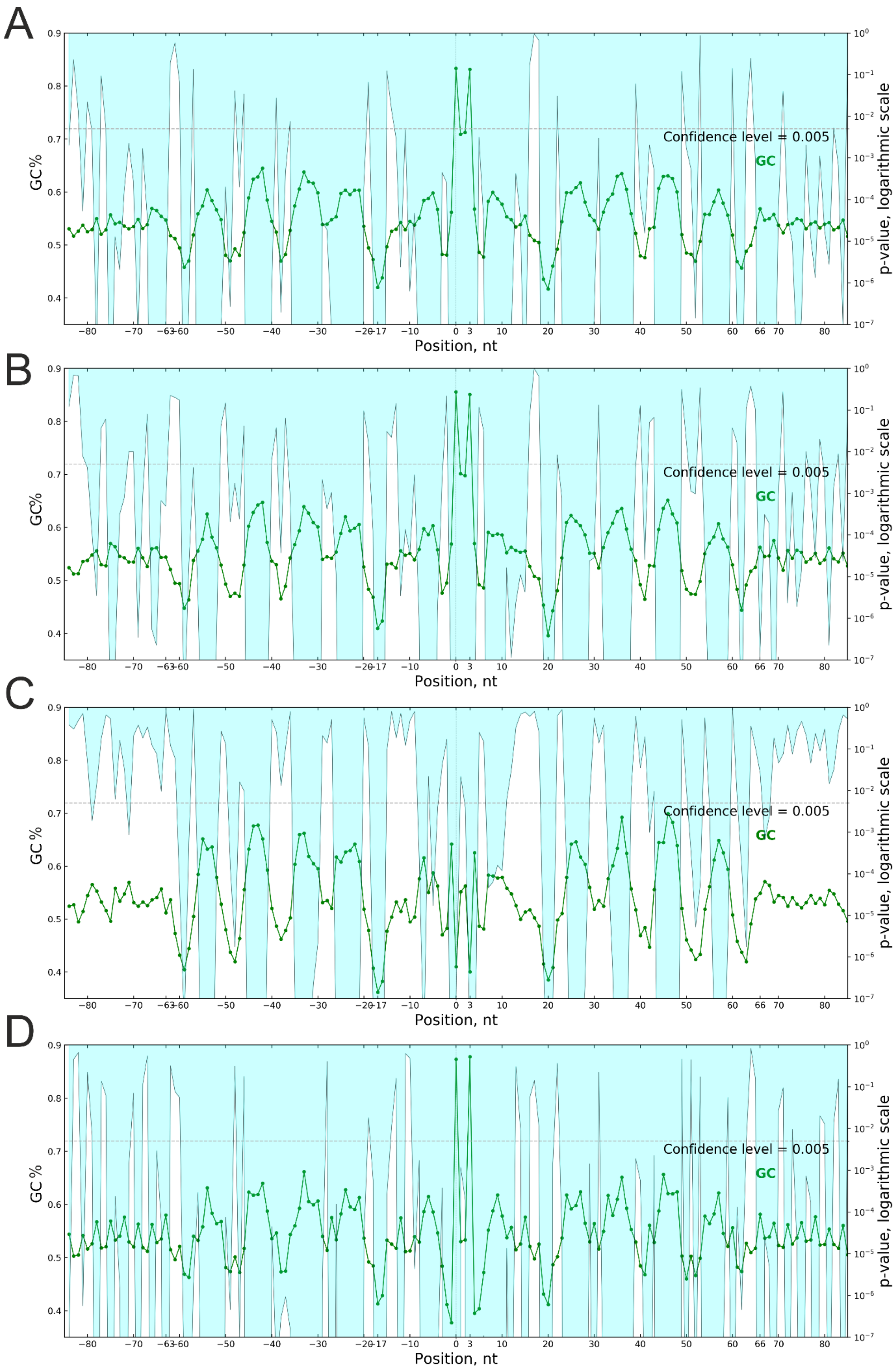


Supplementary Figure S4. **DNA gyrase has a degenerate and long periodic binding motif**. (**A**) Motif found in the Cfx Topo-Seq. (**B**) Motif found in the RifCfx Topo-Seq. (**C**) Motif found in the Micro Topo-Seq. (**D**) Motif found in the Oxo Topo-Seq.

Statistical significance of the fluctuations is shown as light blue filling – contour of the filling is a p-value, calculated for the GC pairs' frequency observed in a particular column of a multiple alignment. The null hypothesis is that the frequency is equal to the frequency of GC pairs in *E. coli* W3110 Mu SGS genome, statistical model – binomial distribution, number of successes – number of G or C in a column of the multiple alignment, total number of events – number of sequences in the alignments. Cfx – 4635 sequences, Rif Cfx – 2355, Micro – 732, Oxo – 5478 sequences. p-value axis is shown on the right in a logarithmic scale. 0.005 confident interval is indicated as a dashed line.

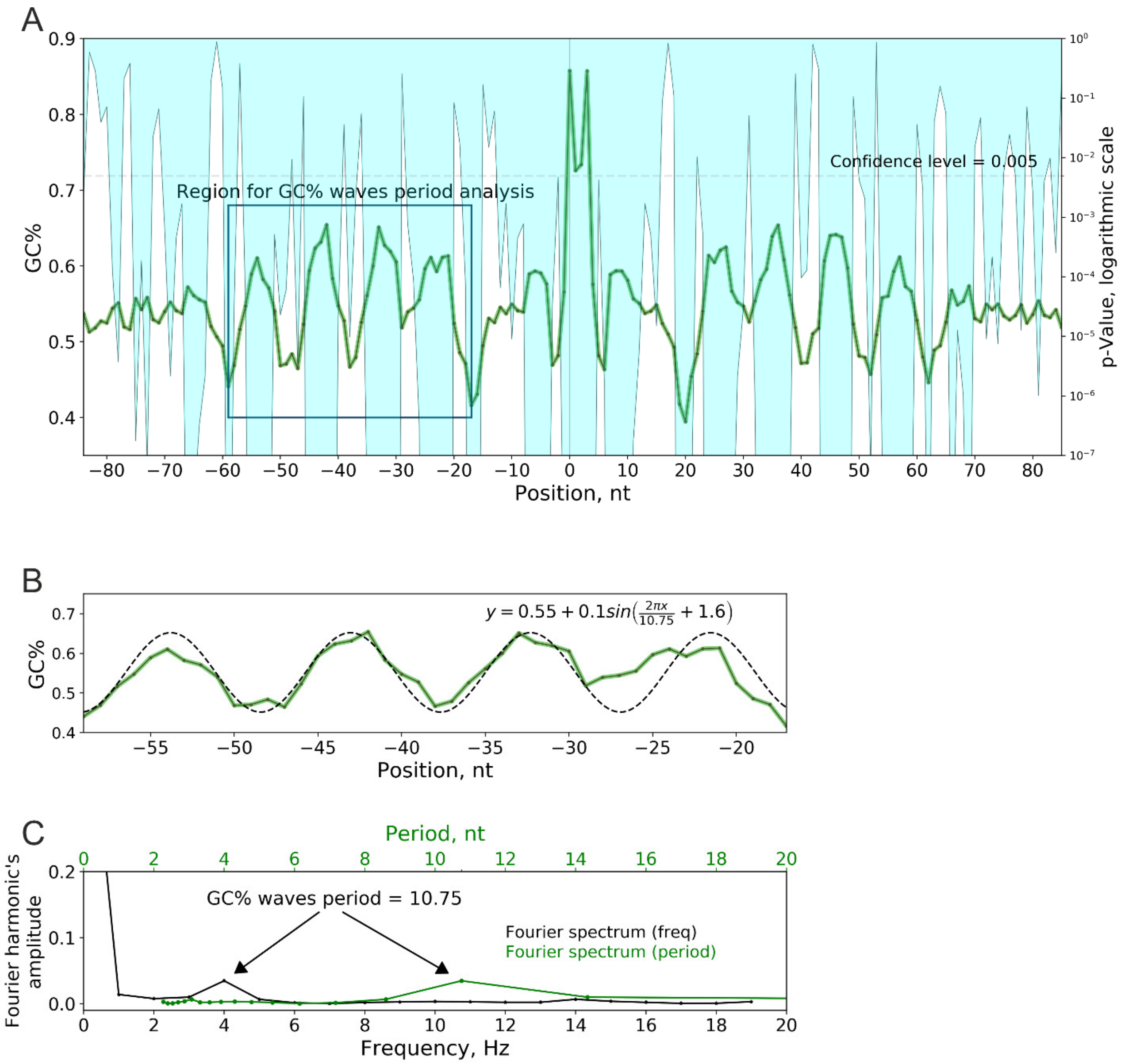


Supplementary Figure S5. **DNA gyrase binding motif periodicity analysis**. (**A**) Overview of the binding motif observed in Cfx Topo-Seq experiment; frequency analysis area is highlighted with a rectangle. Statistical significance of the fluctuations is shown as light blue filling – contour of the filling is a p-value, calculated for the GC pairs' frequency observed in a particular column of a multiple alignment. The null hypothesis is that the frequency is equal to the frequency of GC pairs in *E. coli* W3110 Mu SGS genome, statistical model – binomial distribution, number of success – number of G or C in a column of the multiple alignment, total number of events – number of sequences in the alignment (4635). P-value axis is shown on the right in a logarithmic scale. 0.005 confident interval is indicated as a dashed line (**B**) Fitting of the motif GC% with a *sin* function shown as a dashed black line. (**C**) Fourier spectrum for the frequency analysis area. Spectrum in frequency coordinates shown in black, spectrum in the coordinates of the period – in green; local maxima of the spectra (4 Hz or 10.75 bp, that corresponds to each other) is marked with arrows

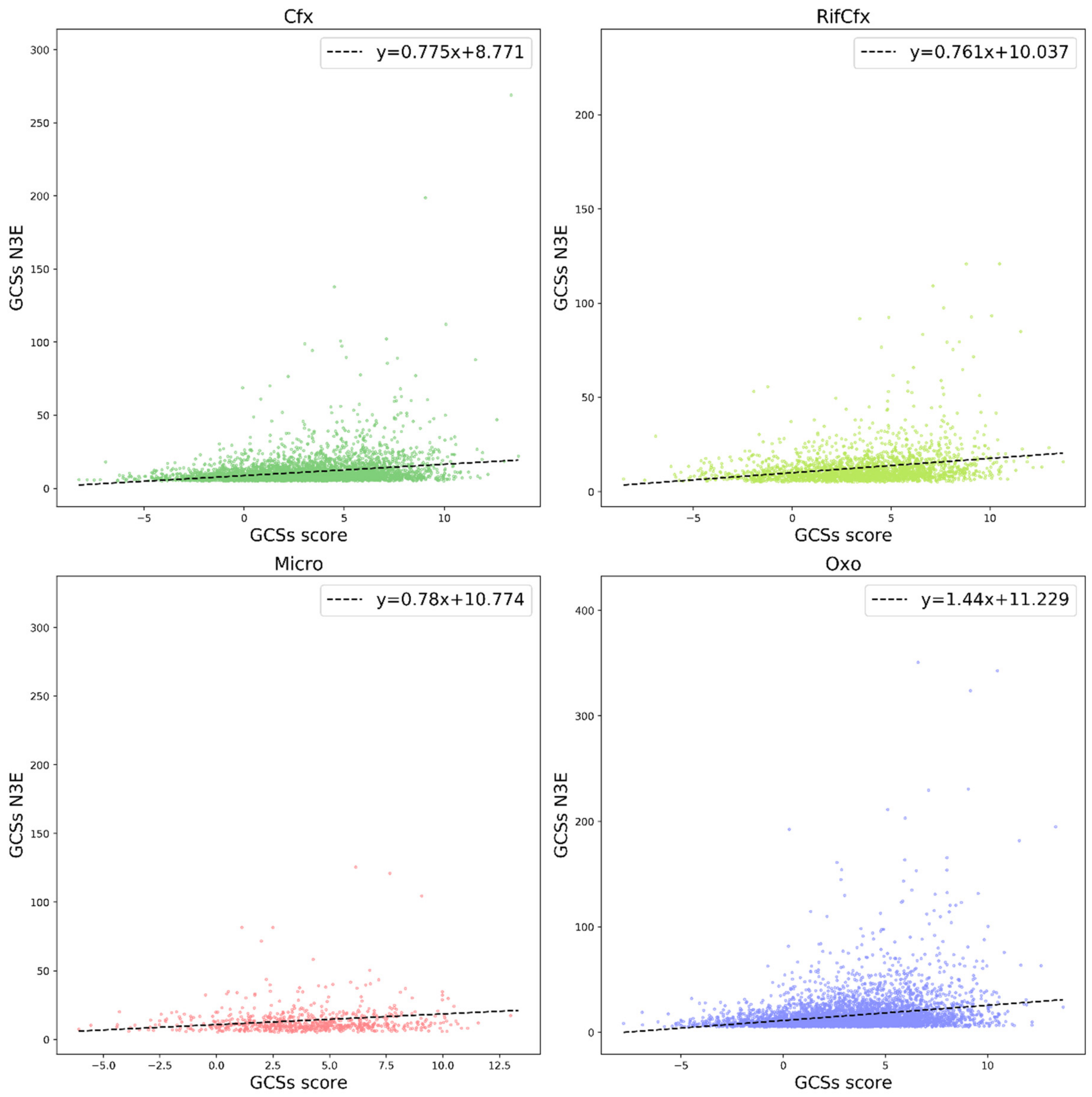


Supplementary Figure S6. **Dependency of GCSs height (N3E value) from sequence score for different Topo-Seq experiments**. Dashed lines indicate linear regressions, equations for which are located on the top left of plots. Regressions were build with Python package numpy.polyfit

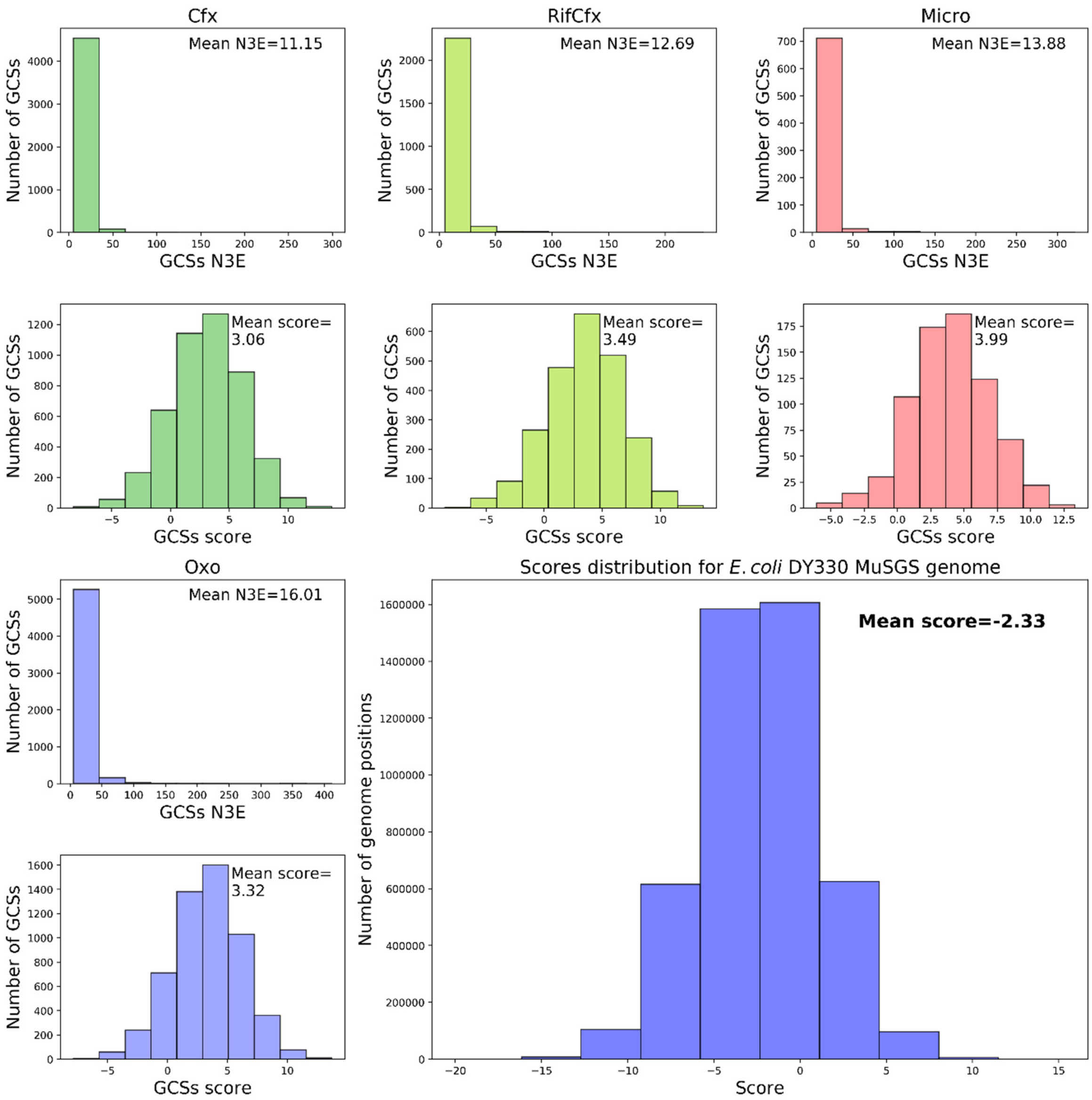


Supplementary Figure S7. **Comparison of GCSs sets obtained in all Topo-Seq experiments (Cfx, RifCfx, Micro, and Oxo)**. For each GCSs set distributions of N3E values and scores are shown. Additionally, distribution of scores for *E. coli* W3110 Mu SGS genome is shown. Mean values are indicated on plots

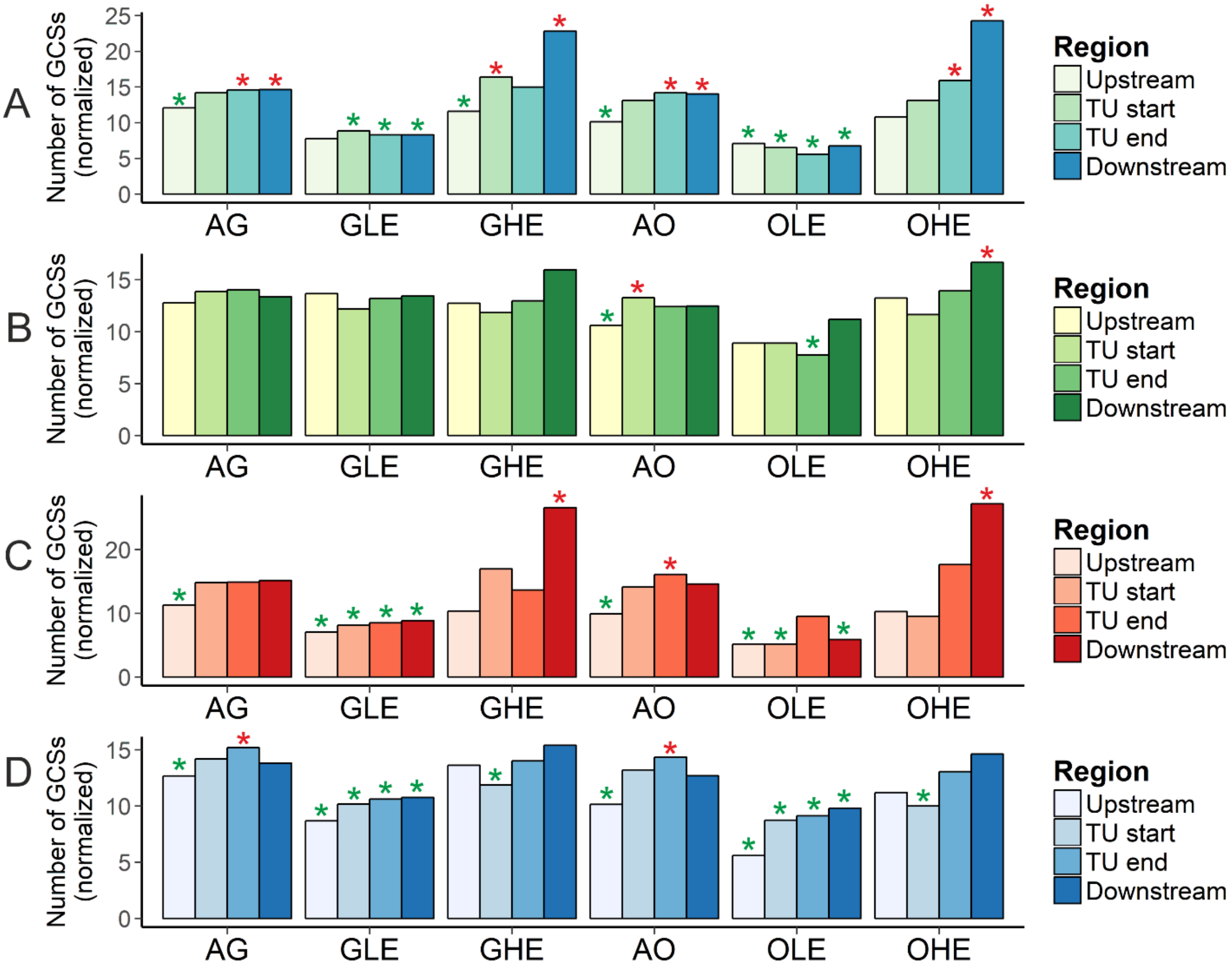


Supplementary Figure S8. **GCSs are associated with highly transcribed genes and accumulate at the ends of genes and downstream (DS) regions**. (**A**) Ciprofloxacin-mediated Topo-Seq. **AG** – all genes were analyzed (4240 genes), **GLE** – genes with low transcription level (379 genes with transcription level less than 0.95 units), **GHE** – genes with high transcription level (379 genes with transcription level more than 100 units), **AO** – all operons were analyzed (2330 operons), **OLE** – operons with low transcription level (194 operons with transcription level less than 1.36 units), **OHE** – operons with high transcription level (194 operons with transcription level more than 101 units). **Upstream** – upstream region, **TU start** – beginning of a transcription unit, **TU end** – end of a transcription unit, **Downstream** – downstream region. All regions have a constant length – 650 bp. Stars above the bars indicate statistically significant deviations in the number of GCSs (p-value<0.01). **Green stars** – regions with significantly lower GCSs than expected, **red stars** – significantly higher number of GCSs.

(**B**) Ciprofloxacin Topo-Seq after Rif treatment. Labelling is the same as above. (**C**) Micro Topo-Seq. (**D**) Oxo Topo-seq

To compare data for different antibiotics and genes sets number of GCSs for each case (antibiotic, TUs set, region) was normalized according to the formula $n_{norm} = \frac{n*100000}{N*G}$, where $n_{norm}$ – normalized number of GCSs, n – number of GCSs observed, N – total number of GCSs for the particular antibiotic, G – number of TUs in the set, 100000 – normalization constant.

Statistical test we used is based on the assumption that GCSs are distributed uniformly and the expected number of GCSs for a particular TUs set is proportional to the number of TUs in the set considered. Particular number of GCSs are tested with binomial test: number of successes is a number of GCSs, total number of events is an expected number of GCSs, probability of the success 0.25 (GCSs are expected to be distributed uniformly between four equal compartments – upstream, TU start, TU end and downstream).

Annotation of genes and operons for *E. coli* W3110 was obtained from DOOR database (NC_007779(C)). Raw data for the number of GCSs associated with compartments was obtained with Genome_intevals_analysis.py, barplots were constructed with GCSs_association_with_TUs_USUS_USGB_GBDS_DSDS_barplot.R

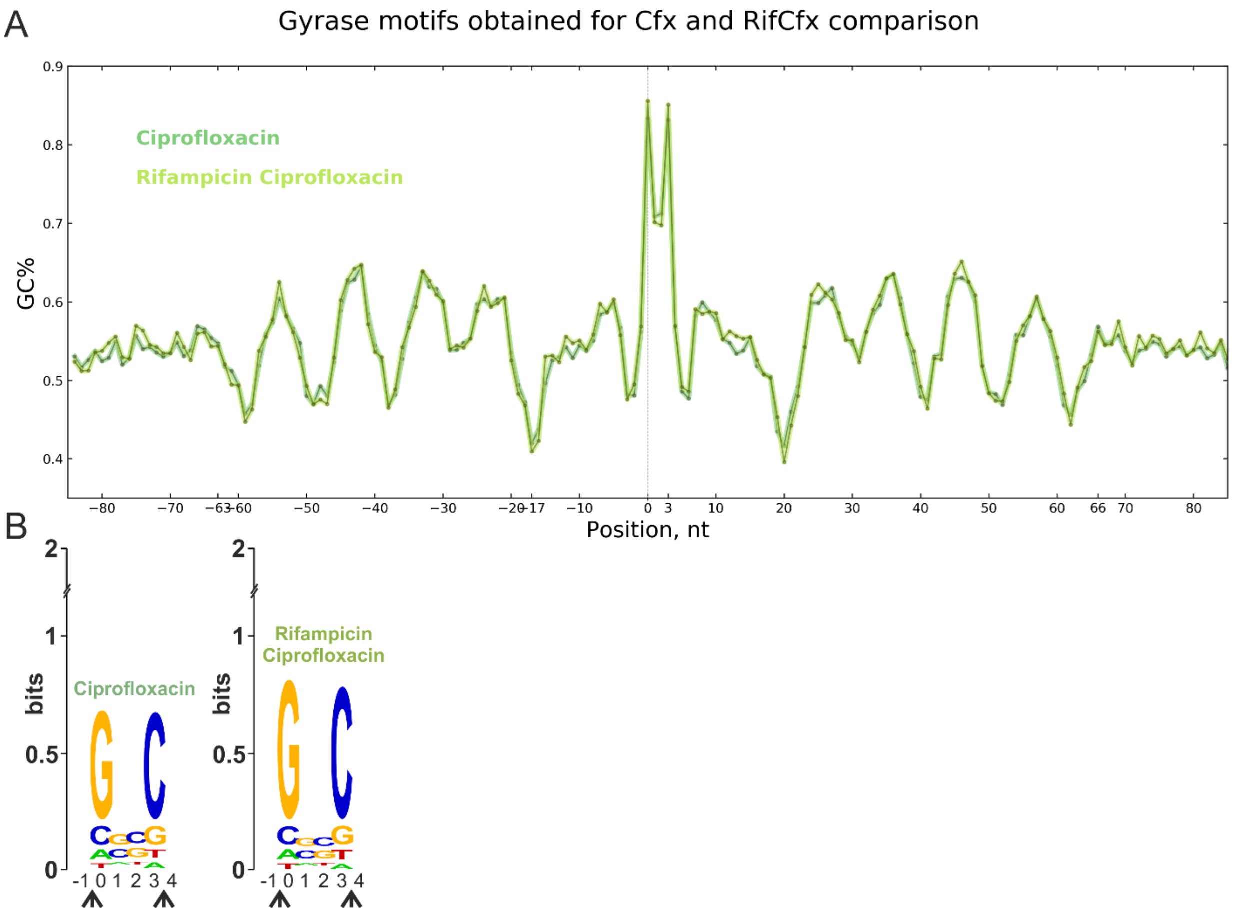


Supplementary Figure S9. **Comparison of DNA gyrase binding motifs obtained when Cfx and RifCfx conditions.** (A) DNA sequences under GCSs were extracted and aligned; resulting in extensive and symmetrical motif, shown as a plot of GC content, has a central region (-16:19 nt) containing cleavage site and two periodic regions (-63:-17 and 20:66 nt). (B) Logo representation of motif's central part around cleavage site. In the coordinates we use, DNA gyrase cleaves forward chain between -1 and 0 and reverse chain between 3 and 4 bp (cleavage events are indicated with arrows)

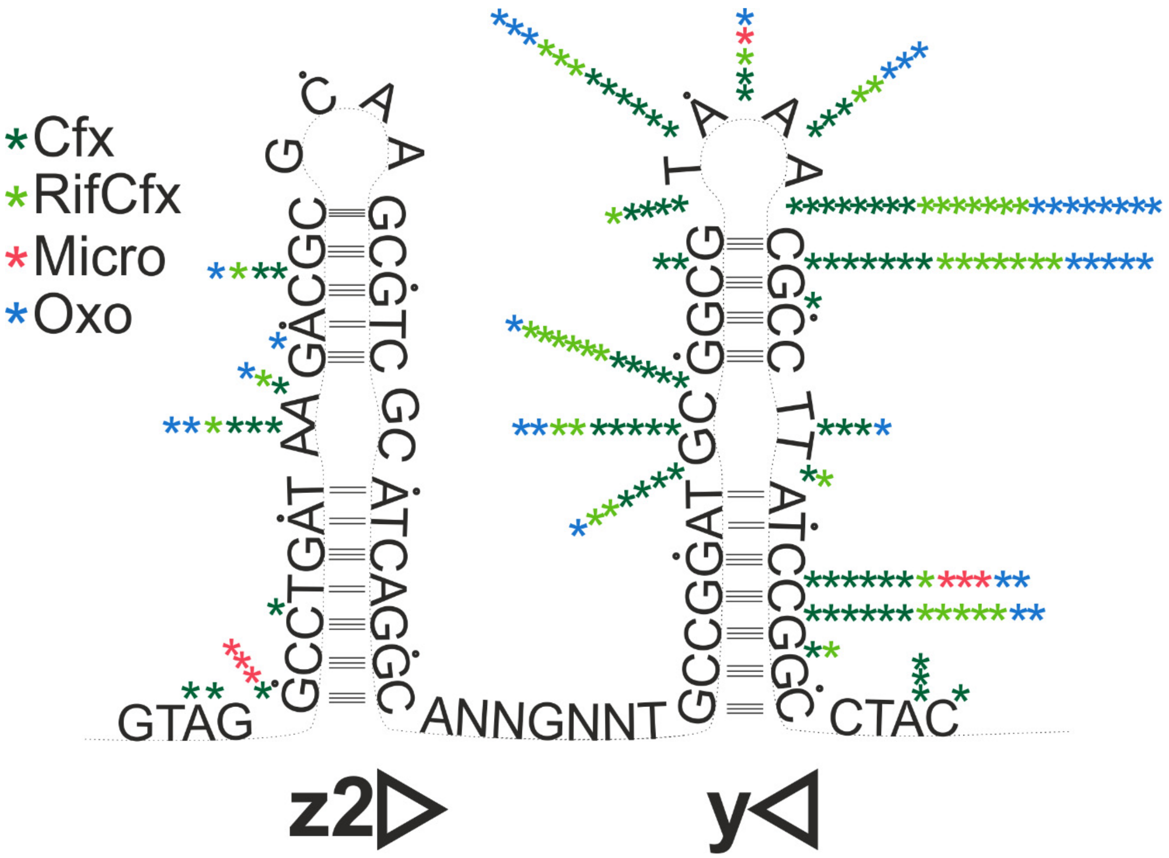


Supplementary Figure S10. **Distribution of gyrase-mediated cleavage events within schematic BIME-2 that consists of one z2 and one y REP**. 13 BIME-2s that contains at least 3 GCSs were annotated with REPs locations and types (**Supplementary Table DS5**). GCSs observed were positioned within these regions and schematic BIME-2 was constructed as a pair of convergent z2 and y REPs. REPs are shown as cruciforms, GCSs indicated as stars, color of stars corresponds to Topo-Seq condition when GCS was observed.

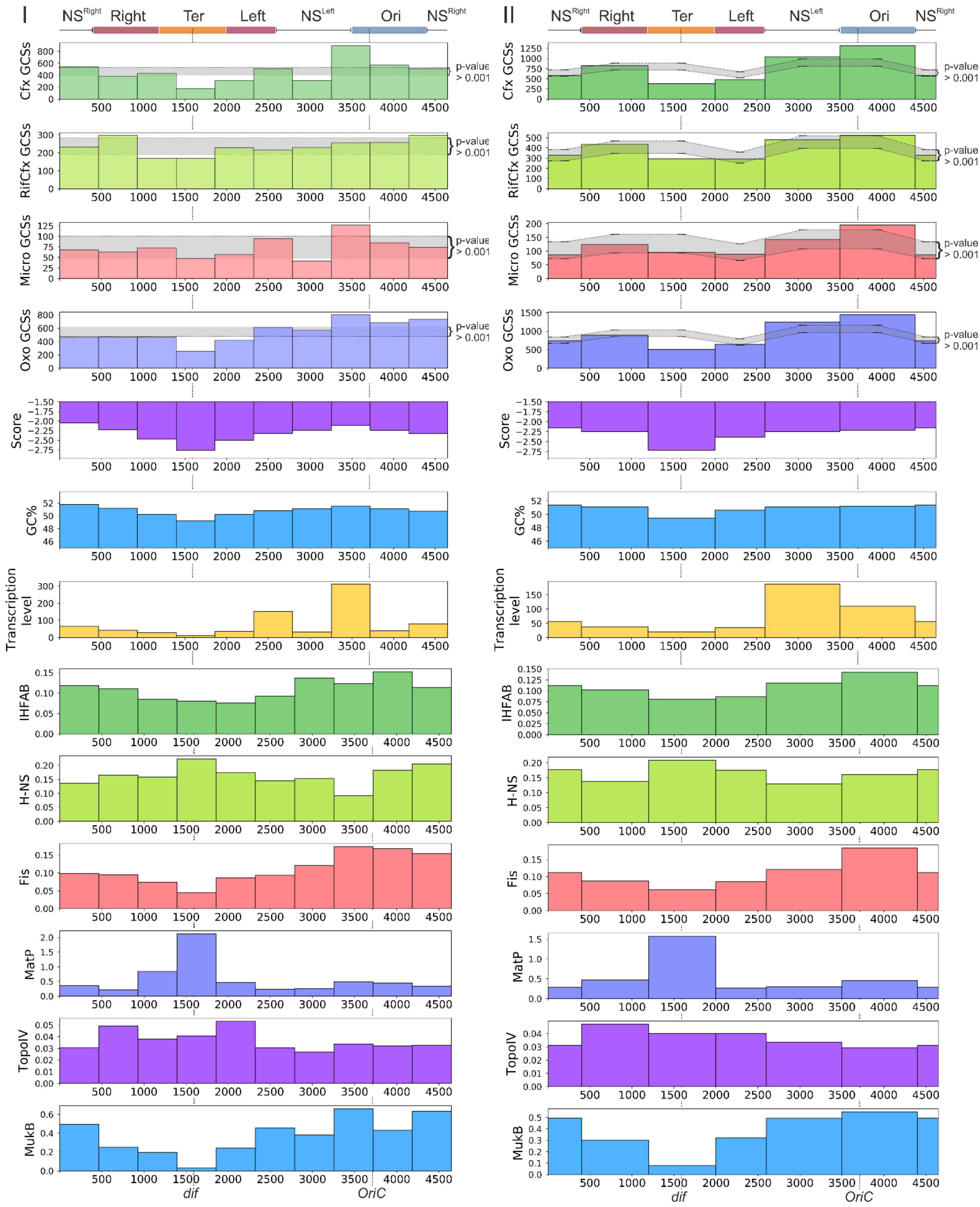


Supplementary Figure S11. **Global distribution of DNA gyrase, sequence score, transcription activity, and NAPs binding**. **I.** On all the plots beneath genome was split into 10 non overlapping bins and for each bin corresponding parameter was calculated. **II.** Genome was split into chromosomal macrodomains according to Valens et al., 2004 (1) and their boundaries were corrected for *E. coli* W3110 Mu SGS as discussed in Duigou, 2017 (2). (**A-D**) Distribution of GCSs revealed by Topo-Seq in the presence of Cfx, Rif and Cfx (RifCfx), Micro, and Oxo respectively. 0.999 confidential interval is shown as a light grey zone,

number of GCSs in each bin set to be binomial (null hypothesis - GCSs are distributed uniformely with the probability of GCS to fall into particular bin to be proportional to the width of the bin). (**E**) Gyrase binding motif score calculated for *E. coli* W3110 Mu SGS genome. (**F**) GC% of *E. coli* W3110 Mu SGS genome. (**G**) Transcription level over *E. coli* W3110 Mu SGS genome calculated as a coverage depth for RNA-Seq experiment (3). (**H**) IHF binding over the genome (binding sites detected for both IHF A and IHF B polypeptides), data taken from Prieto et al, 2012 (4). (**I**) H-NS binding over the genome, data taken from Kahramanoglou et al, 2010 (5). (**J**) Fis binding over the genome, data taken from Kahramanoglou et al, 2010 (5). (**K**) MatP binding over the genome, data taken from Nolivos et al., 2016 (6). (**L**) TopoIV cleavage over the genome, data taken from Sayyed at al., 2016 (7). (**M**) MukB binding over the genome, data taken from Nolivos et al., 2016 (6).

For IHFAB, H-NS, and Fis y-axis indicates a proportion of a genomic bin, which is covered with corresponding binding sites. For MatP, TopoIV, and MukB this proportion is weighted using fold enrichment data available for every binding region of these NAPs (for details see Topo-seq project on GitHub script GCSs_transcription_score_GC_distributions_throughout_genome.py).

**NS$^{Right}$** – right non-structured region; **Right** – right macrodomain; **Ter** – terminator domain; **Left** – left macrodomain; **NS$^{Left}$** – left non-structured region; **Ori** – origin domain. Positions of *OriC* and *dif* are indicated by dashed marks. Transcription data obtained from Incarnato et al., 2017 (3)

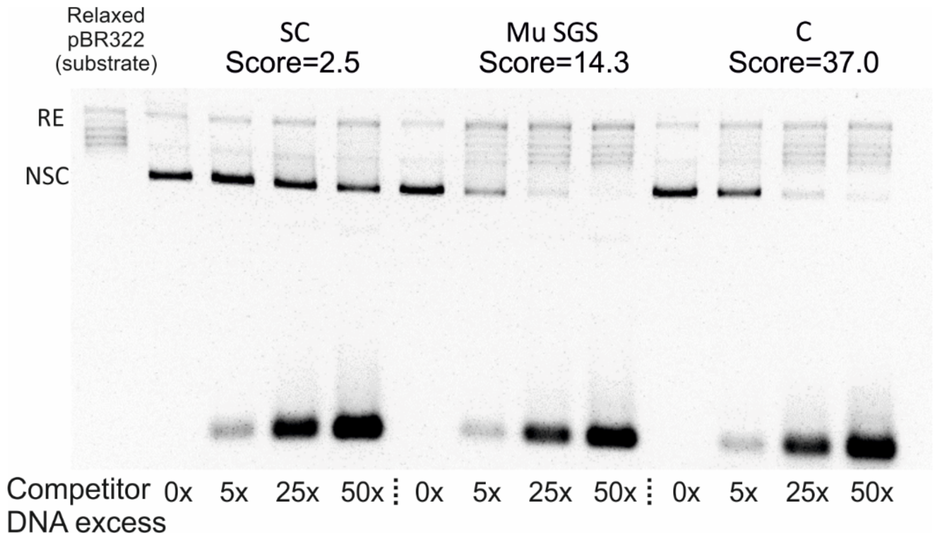


Supplementary Figure S12. **Competition between relaxed pBR322 plasmid and 133 bp linear DNA fragments having different score for interaction with DNA gyrase**. SC - scrambled consensus, Mu SGS - strong gyrase binding site from bacteriophage Mu, C - consensus sequence. RE - relaxed state of the pBR322 plasmid, NSC - negatively supercoiled state of the plasmid.

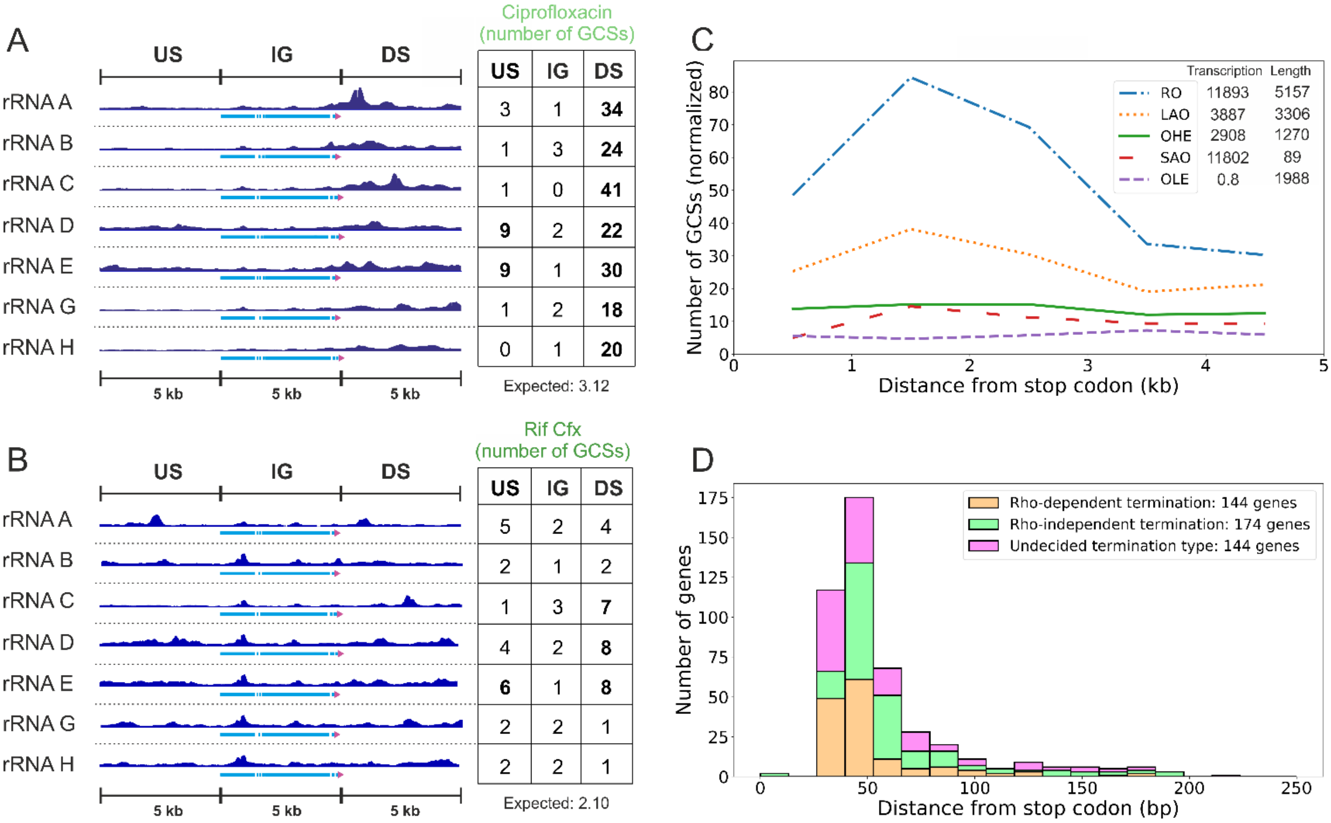


Supplementary Figure S13. **Association of GCSs with downstream regions of active and long operons**. (**A**) Enrichment of GCSs in downstream (DS) regions of rRNA operons revealed by Topo-Seq with Cfx. Coverage depth of the regions containing operons are shown on the left. Amounts of GCSs in upstream (US), operon bodies (IG) and DS are summarized in the table on the right. Numbers that are significantly exceeded expected values are in bold (binomial test, p-value<0.01). (**B**) The same as in (A) for RifCfx Topo-Seq. (**C**) Localization of GCSs (GCSs observed in any of the Topo-Seq conditions) in DS regions of operons. RO - rRNA operons (7 operons), LAO - long and active operons (27 operons), OHE - operons with high transcription level (186 operons), SAO - short and active operons (27 operons), OLE - operons with low transcription level (186 operons). For each set of operons mean transcription level and mean length are indicated. Number of GCSs was normalized on the number of operons and the total number of GCSs. (**D**) Distribution of distances from gene stop codon to the actual site of transcription termination for different types of transcription terminators: Rho-dependent (144 genes), Rho-independent (174 genes), or of undecided type (144 genes). Data from Dar & Sorek, 2018 (8).

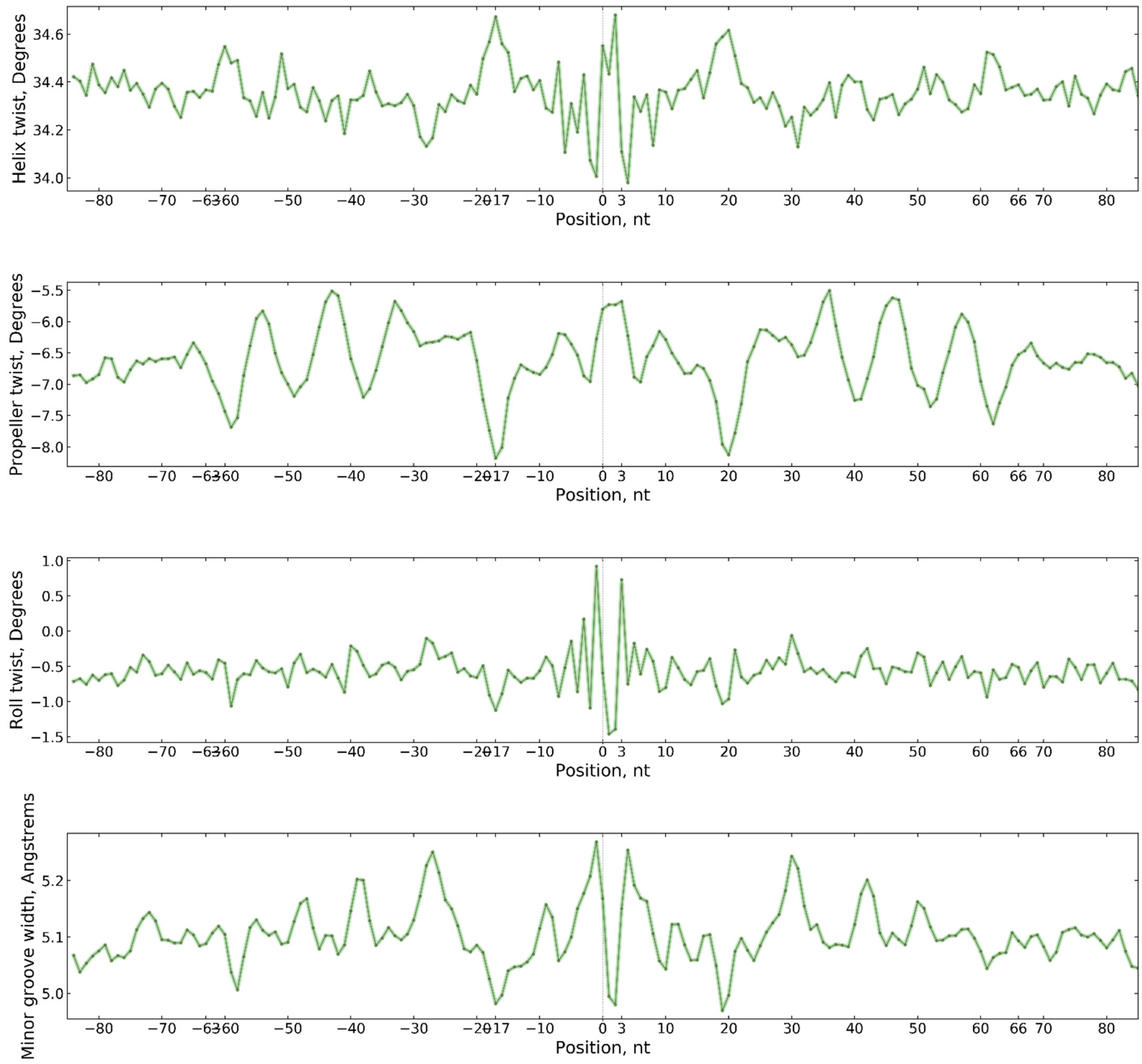


Supplementary Figure S14. **DNA geometry underlies indirect readout of DNA by the DNA gyrase**. Several DNA geometry parameters (helix twist, propeller twist, roll twist, minor groove twist) were calculated for 1828 sequences used to construct a “combined motif”. Geometry data were obtained from GBshape database (9). Central part of the motif was not de-biased at drug-dependent positions (0-3 bp).

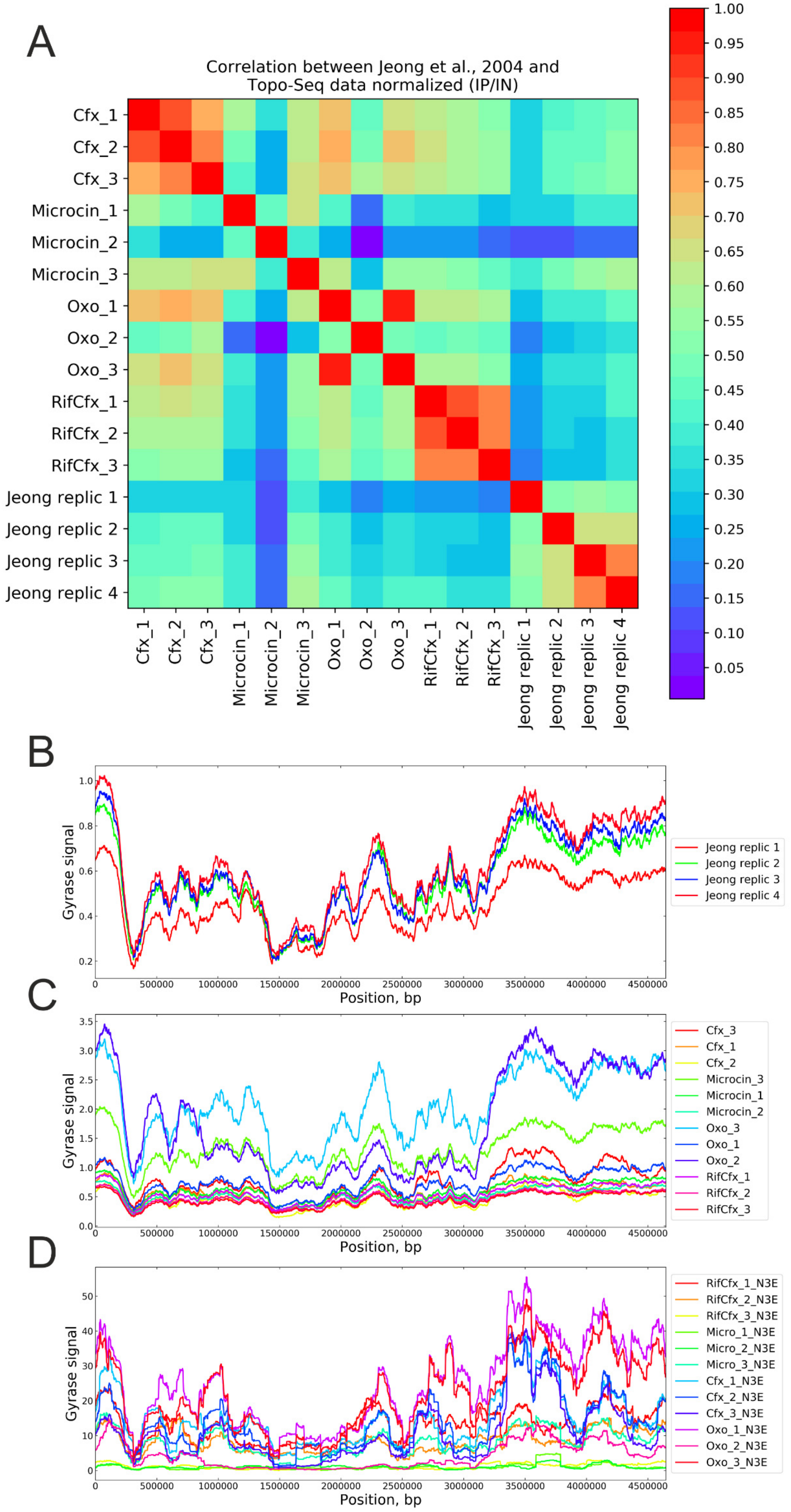


Supplementary Figure S15. **Correlation and correspondence between gyrase binding data (Jeong et al., 2004** (10)**) and Topo-Seq data**. (**A**) Heatmap represents the Pearson correlation coefficients calculated between 4 replicas of Jeong et al., 2004 gyrase binding data and all replicas of Topo-Seq. (**B**) Binding of DNA gyrase to the *E. coli W3110* chromosome according to Jeong et al., 2004 smoothed with a 200-kb sliding window. (**C**) Coverage depth over the *E. coli W3110* genome in Topo-Seq experiments, smoothed with a 200-kb sliding window. (**D**) DNA gyrase cleavage data across *E. coli W3110* genome, smoothed with a 200-kb sliding window.

Details of data analysis are described on the next page.

The analysis was performed on a set of 2315 genes, which were found to have common names for both Jeong et al., 2004 data annotation (Supplementary data, GyrA binding in WT, annotation for *E. coli* K-12 MG1655) and *E. coli W3110* genome annotation we use.

- Jeong et al., 2004 ChIP-chip data have gene resolution and represent a ratio of gyrase IP DNA signal to genomic DNA signal for each gene.
- Topo-Seq raw coverage depth data have a single-base resolution and obtained for gyrase IP and mock DNA separately.
- Topo-Seq GCSs data represent single-base signals (correspond to gyrase cleavage sites), which are already normalized on signals from mock control.

To make Topo-Seq raw coverage depth data comparable with data by Jeong et al., firstly, they were gene-binned by calculating mean coverage depth for each gene. Secondly, values obtained for IP sample were divided by corresponding values for IN samples. Obtained ratios were correlated with Jeong's data, giving heatmap (**A**).

Similarly, Topo-Seq GCSs data was also gene-binned - a gene signal was calculated as a sum of GCSs' N3Es which fall within a gene.

Genome tracks (**B**)-(**D**) were constructed with equal-format data as following:
1) Gene-binned data was deconvoluted back to get some signal for every genome position. For this, genome positions covered with a gene having a signal got a signal equal to the overall gene signal.
2) Gene order was set as in *E. coli W3110*.
3) Data obtained was smoothed by averaging sliding window 200 kb wide.

Thus, "Gyrase signal" for the plot (**B**) is an averaged ratio between gyrase IP and mock DNA chip signals for genes. "Gyrase signal" for plot (**C**) is an averaged ratio between gyrase IP and mock DNA coverage depths for genes. "Gyrase signal" for plot (**D**) is an averaged gyrase cleavage signal calculated for genes.

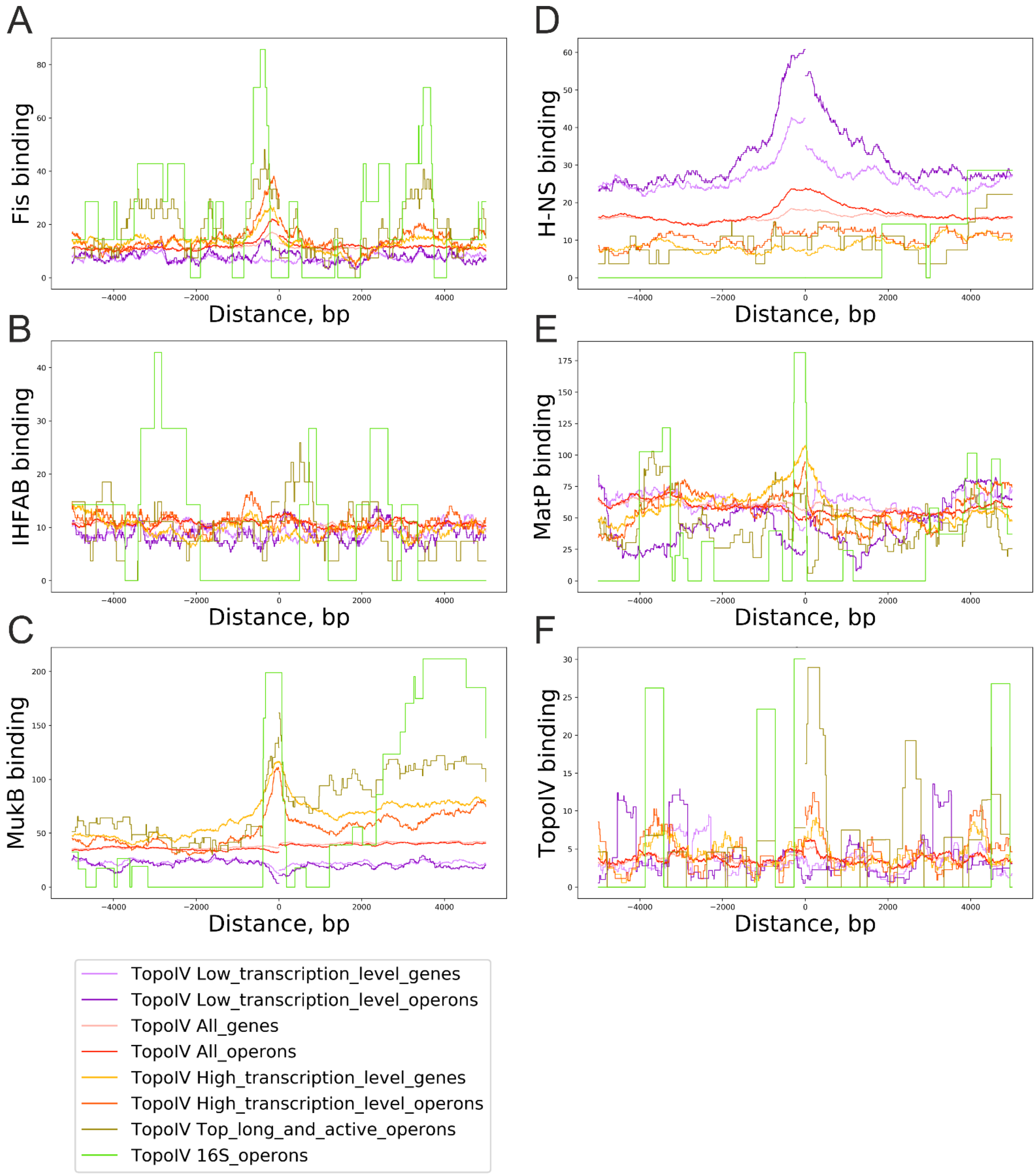


Supplementary Figure S16. **Association of NAPs with upstream and downstream regions of transcriptional units**. Start and stop codons correspond to position 0. Upstream region is on the left of 0, downstream is on the right. TUs sets which were used in the analysis are the same as for Figure S8 and Figure S13: All genes (4121 TUs); All operons (2328 TUs); High transcription level genes (370 TUs); High transcription level operons (186 TUs); Low transcription level genes (370 TUs); Low transcription level operons (186 TUs); Top long and active operons (27 TUs); 16S operons (7 TUs). Upstream and downstream regions were aligned relative to the position of start and stop codons, respectively, and an overall signal was calculated. Signal was divided on the number of TUs in a set and multiplied by 100.

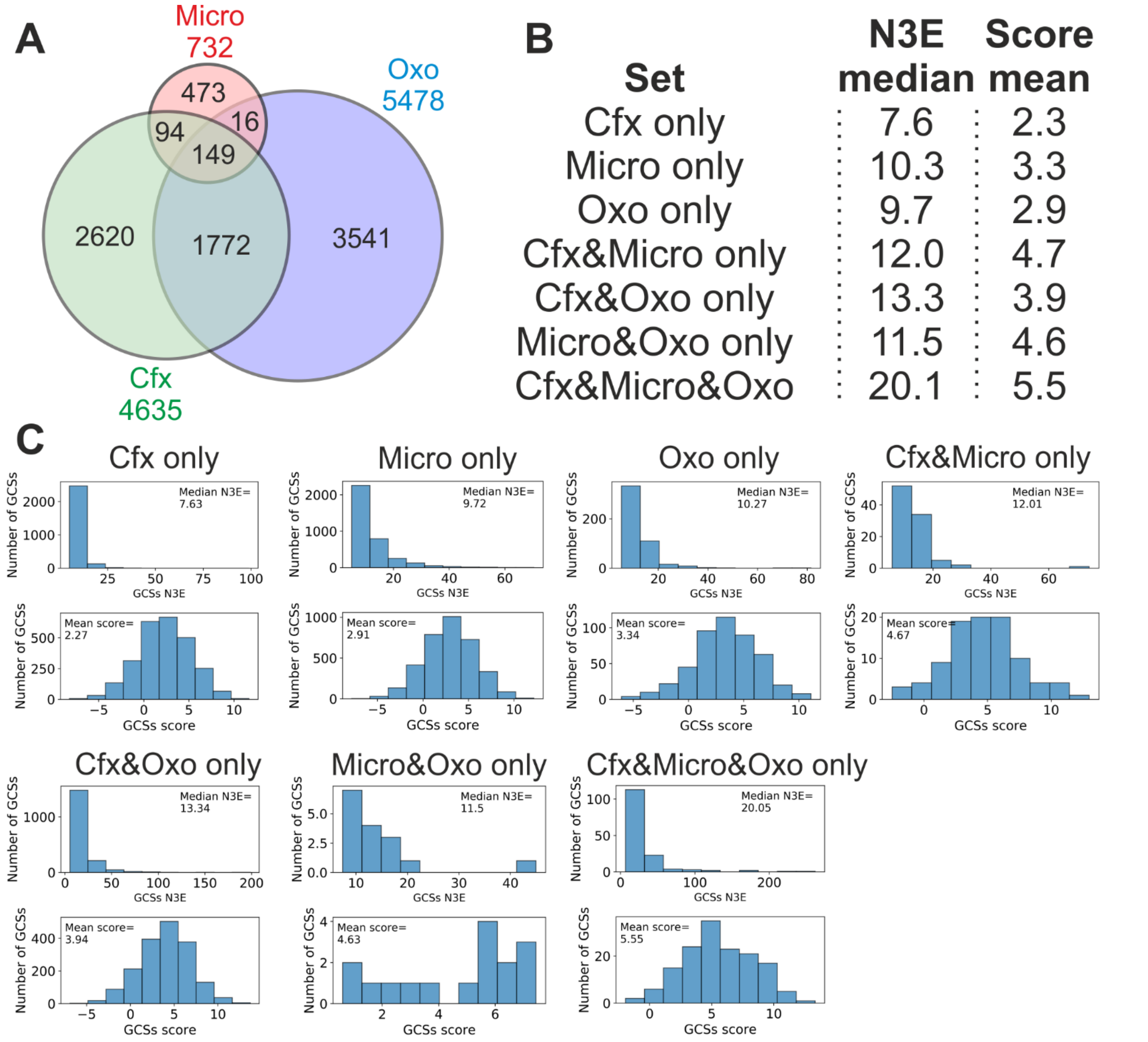


Supplementary Figure S17. **Cleavage sites simultaneously revealed by several poisons have higher signals (N3E) and have high scores**. (**A**) Venn diagram representation of the relations between GCSs sets obtained with different gyrase poisons (same as **Fig. 3A**). (**B**) Comparison of N3E medians and score means for different subsets of GSCs. (**C**) Distributions of N3E and score for different subsets of GCSs.

REFERENCES


1. Valens M., Penaud S., Rossignol M., Cornet F. and Boccard F. (2004) Macrodomain organization of the Escherichia coli. *EMBO J.*, **23**, 4330–4341.

2. Duigou S. and Boccard F. (2017) Long range chromosome organization in Escherichia coli: The position of the replication origin defines the non-structured regions and the Right and Left macrodomains. *PLoS Genet.*, **13**.

3. Incarnato D., Morandi E., Anselmi F., Simon L.M., Basile G. and Oliviero S. (2017) In vivo probing of nascent RNA structures reveals principles of cotranscriptional folding. *Nucleic Acids Res.*, **45**, 9716–9725.

4. Prieto A.I., Kahramanoglou C., Ali R.M., Fraser G.M., Seshasayee A.S.N. and Luscombe N.M. (2012) Genomic analysis of DNA binding and gene regulation by homologous nucleoid-associated proteins IHF and HU in Escherichia coli K12. *Nucleic Acids Res.*, **40**, 3524–3537.

5. Kahramanoglou C., Seshasayee A.S.N., Prieto A.I., Ibberson D., Schmidt S., Zimmermann J., Benes V., Fraser G.M. and Luscombe N.M. (2011) Direct and indirect effects of H-NS and Fis on global gene expression control in Escherichia coli. *Nucleic Acids Res.*, **39**, 2073–2091.

6. Nolivos S., Upton A.L., Badrinarayanan A., Muller J., Zawadzka K., Wiktor J., Gill A., Arciszewska L., Nicolas E. and Sherratt, D. (2016) MatP regulates the coordinated action of topoisomerase IV and MukBEF in chromosome segregation. *Nat. Commun.*, **7**.

7. El Sayyed, H., Le Chat, L., Lebailly, E., Vickridge, E., Pages, C., Cornet, F., Cosentino Lagomarsino, M. and Espéli, O. (2016) Mapping Topoisomerase IV Binding and Activity Sites on the E. coli Genome. *PLoS Genet.*, **12**, 1–22.

8. Dar D. and Sorek R. (2018) High-resolution RNA 3 -ends mapping of bacterial Rho-dependent transcripts. *Nucleic Acids Res.*, **46**, 6797–6805.

9. Chiu T., Yang L., Zhou T., Main B.J., Parker S.C.J., Nuzhdin V., Tullius T.D. and Rohs R. (2015) GBshape : a genome browser database for DNA shape annotations. *Nucleic Acids Res.*, **43**, 103–109.

10. Jeong K.S., Ahn J. and Khodursky A.B. (2004) Spatial patterns of transcriptional activity in the chromosome of Escherichia coli. *Genome Biol.*, **5**, R86.
